# Graph-guided adaptive companding for PAPR reduction in power-domain NOMA systems

**DOI:** 10.1371/journal.pone.0349671

**Published:** 2026-05-21

**Authors:** Arun Kumar, Mehedi Masud, Mansor Alohali, Prashanta Chandra Pradhan, Aziz Nanthaamornphong

**Affiliations:** 1 Department of Electronics and Communication Engineering, Sikkim Manipal Institute of Technology, Sikkim Manipal University, Majitar, Rangpo, Sikkim, India; 2 Department of Computer Science, College of Computers and Information Technology, Taif University, Taif, Saudi Arabia; 3 Applied College, Imam Mohammad Ibn Saud Islamic University (IMSIU), Riyadh, Saudi Arabia; 4 Department of Computer Science & Engineering, Sikkim Manipal institute of technology, Sikkim Manipal University, Majitar, Rangpo, Sikkim, India; 5 College of Computing, Prince of Songkla University, Phuket, Thailand; Beijing Technology and Business University, CHINA

## Abstract

Power-domain Non-Orthogonal Multiple Access (PD-NOMA) is a promising multiple-access technique for fifth-generation (5G) and beyond-fifth-generation (B5G/6G) wireless networks because of its ability to improve spectral efficiency, massive connectivity, and user fairness through superposition coding and successive interference cancellation (SIC). However, when combined with multicarrier modulation such as Orthogonal Frequency Division Multiplexing (OFDM), PD-NOMA suffers from a high Peak-to-Average Power Ratio (PAPR), which degrades the power amplifier efficiency, increases nonlinear distortion, and significantly impairs the bit error rate (BER) and SIC reliability. Existing PAPR reduction techniques, particularly conventional companding schemes, apply uniform or globally adaptive nonlinear transformations and fail to account for the inherent multiuser coupling and SIC sensitivity of PD-NOMA signals. To address these limitations, this study proposes a novel Graph-Guided Adaptive Companding (GGAC) framework for PD-NOMA systems. The proposed method models the composite PD-NOMA waveform as a graph, where the signal components are represented as nodes, and their power and interference relationships are captured through weighted edges. Graph-based importance metrics are then used to assign node-specific companding parameters, enabling the selective suppression of peak-dominant components while preserving the integrity of low-power and SIC-critical users. The simulation results demonstrate that GGAC achieves a significant PAPR reduction, offering gains of up to 10 dB over conventional PD-NOMA. In addition, the proposed framework significantly improves the BER and Signal-to-Interference-plus-Noise Ratio (SINR), reduces out-of-band radiation by more than 40 dB, and preserves the constellation geometry with a lower error vector magnitude. These results confirm that GGAC provides an effective, low-complexity, and scalable solution for enhancing the energy efficiency and reliability in future PD-NOMA-based 5G systems.

## Introduction

The rapid evolution of wireless communication systems toward 5G and beyond-5G (B5G/6G) networks has been driven by unprecedented demands for massive connectivity, ultrahigh data rates, low latency, and enhanced energy efficiency. Emerging applications such as massive Internet of Things (mIoT), ultra-reliable low-latency communications (URLLC), autonomous systems, smart healthcare, and immersive extended reality require flexible and scalable multiple access schemes capable of supporting heterogeneous users with diverse quality-of-service (QoS) requirements [1]. In this context, power-domain Non-Orthogonal Multiple Access (PD-NOMA) has been recognized as a key technology for improving spectral efficiency, user fairness, and connectivity in 5G and B5G networks. By allowing multiple users to share the same time–frequency resources through superposition coding at the transmitter and successive interference cancellation (SIC) at the receiver, PD-NOMA overcomes the rigid resource partitioning limitations of conventional orthogonal multiple access (OMA) schemes [2]. PD-NOMA significantly enhances the performance of 5G and beyond-5G systems in several fundamental manners. First, it improves spectral efficiency by multiplexing multiple users on the same resource block, which is particularly beneficial in dense networks with limited spectrum availability. Second, PD-NOMA supports massive connectivity by enabling a large number of devices, including low-power IoT nodes, to simultaneously access the network. Third, by allocating different power levels to users based on their channel conditions, PD-NOMA enhances fairness, allowing cell-edge users with poor channel quality to be reliably served along with users with strong channels. These advantages make PD-NOMA a strong candidate for integration with advanced 5G technologies, such as massive MIMO, millimeter-wave communications, and cooperative relay networks, and for future B5G/6G paradigms emphasizing intelligent, adaptive, and user-centric communication [3]. Despite its promising benefits, the practical implementation of PD-NOMA faces significant physical layer challenges, among which the Peak-to-Average Power Ratio (PAPR) problem is particularly critical. When PD-NOMA is combined with multicarrier modulation techniques such as Orthogonal Frequency Division Multiplexing (OFDM), which are widely adopted in 5G systems owing to their robustness against multipath fading, the superposition of multiple user signals with different power levels results in large signal amplitude fluctuations. A high PAPR forces power amplifiers to operate with a large back-off to avoid nonlinear distortion, leading to reduced power efficiency, increased energy consumption, spectral regrowth, and degradation in the bit error rate (BER). These issues are particularly detrimental in 5G and B5G networks, where energy efficiency, green communication, and cost-effective hardware design are key objectives. Moreover, nonlinear distortion caused by high PAPR can severely affect the reliability of SIC, thereby undermining the performance gains promised by PD-NOMA [4].

Conventional PAPR reduction techniques, such as clipping and filtering, selective mapping, partial transmit sequences, and transform-domain approaches, have been widely investigated in OFDM systems. Among these methods, companding techniques stand out because of their low computational complexity and absence of side information requirements, making them attractive for real-time and low-latency applications. Companding reduces the PAPR by compressing the high-amplitude signal peaks at the transmitter and expanding them at the receiver. However, traditional companding schemes are typically static and apply uniform nonlinear transformations regardless of the user power level, interference structure, or signal dynamics [5]. In PD-NOMA systems, such blind companding may introduce excessive distortion to weak users, disrupt SIC ordering, and compromise fairness, ultimately limiting achievable performance improvements in 5G and B5G networks. Recent advances in graph-based signal processing have provided a powerful and flexible framework for modeling complex relationships in multiuser communication systems. Graph theory enables the representation of interactions, dependencies, and correlations among users, subcarriers, and signal components through nodes and weighted edges. In PD-NOMA systems, where users are inherently coupled through superposition coding and interference, graph-based models offer an intuitive and mathematically rigorous method to capture the underlying structure of the transmitted signal. By encoding power disparities, interference strength, or correlation metrics into graph topologies, meaningful structural information that can guide intelligent signal processing operations can be extracted [6]. Motivated by these insights, this study proposes a Graph-Guided Adaptive Companding framework for PAPR reduction in power-domain NOMA systems. The proposed approach exploits graph-based representations of the NOMA signal to dynamically adapt the companding parameters according to local and global signal structures. Unlike conventional companding techniques that apply fixed compression characteristics, the graph-guided approach identifies dominant peaks, strongly interfering components, and SIC-critical users through graph metrics, such as node degree, centrality, and edge weights. This enables selective and nonuniform companding, where stronger compression is applied to critical components while preserving the integrity of low-power and edge users.

The proposed framework aligns well with the intelligent and adaptive nature of 5G-Advanced and beyond-5G systems. By jointly addressing PAPR reduction, multiuser interference, and SIC robustness, graph-guided adaptive companding enhances the energy efficiency, improves the BER performance, and supports reliable massive connectivity. Furthermore, the graph-based formulation provides a scalable and extensible foundation that can be integrated with machine learning and optimization techniques, paving the way for intelligent physical layer design in future wireless networks. Using this approach, the practical viability and performance benefits of PD-NOMA can be significantly enhanced, contributing to the realization of efficient, flexible, and high-capacity 5G and beyond-5G communication systems.

## Literature review

Kumar proposed a low-complexity hybrid PTS–SLM–companding technique to reduce the PAPR in 5G NOMA waveforms [7]. This method combines phase rotation (PTS and SLM) with nonlinear companding to suppress high peaks without transmitting side information. Simulation results demonstrate significant PAPR reduction compared to the conventional PTS and SLM methods, with only slight BER degradation. This approach improves power amplifier efficiency and is suitable for practical NOMA-based multicarrier systems. Mounir et al. introduced a low-complexity selective mapping technique specifically designed for downlink OFDM-NOMA systems [8]. The scheme reduces the computational complexity by limiting the phase sequence combinations while maintaining the SIC decoding order. The simulation results show a noticeable PAPR reduction and improved BER performance compared to conventional SLM-based NOMA systems. This study highlights the importance of complexity-aware PAPR reduction to support dense 5G downlink deployment. George and Mishra investigated selective mapping for PAPR reduction in filtered NOMA (F-NOMA) systems [9]. The authors evaluated the CCDF and BER performances under nonlinear power amplifier conditions. The results indicate that SLM effectively reduces PAPR and enhances SIC performance, albeit at the cost of increased computational complexity and side information. This study confirmed the need for low-distortion PAPR reduction techniques for advanced NOMA waveforms. Ramavath and Samal presented a theoretical analysis of various companding techniques applied to FBMC systems [10]. Mathematical expressions for PAPR and signal distortion were derived and evaluated. The results show that exponential and logarithmic companding outperform traditional μ-law companding in terms of PAPR reduction with lower BER penalties. This study emphasizes the necessity of adaptive companding in modern multicarrier systems. Hassan proposed a three-layer hybrid PAPR reduction method for NOMA-based FBMC visible-light communication networks [11]. The scheme integrates precoding, companding, and clipping to reduce peak power while satisfying optical constraints. The simulation results demonstrate superior PAPR reduction and BER performance compared to single-layer techniques. This study highlighted the effectiveness of hybrid and adaptive PAPR solutions in highly nonlinear VLC environments. Hossain et al. introduced a DFT-spread OTFS communication system to mitigate PAPR and nonlinear degradation [12]. DFT spreading redistributes the signal energy across the subcarriers, reducing amplitude fluctuations. The simulation results show a significant PAPR reduction and improved BER under nonlinear HPA conditions. This study demonstrates that structural signal transformation is effective for next-generation waveforms. Chennamsetty et al. analyzed the PAPR performance of OTFS modulation by using classical selective mapping [13]. Different phase sequence lengths were evaluated to balance the performance and complexity. The results showed a moderate PAPR reduction but an increased computational burden. The study concluded that classical SLM alone is insufficient for advanced multiuser systems, motivating hybrid and adaptive approaches. Baena-Lecuyer et al. proposed a low-PAPR preamble-based channel-estimation scheme for OTFS systems [14]. This method designs structured preambles to minimize peak power during synchronization and estimation. The results showed an improved channel estimation accuracy and reduced nonlinear distortion. Although this study focused on preambles, it highlights the importance of structure-aware signal design for PAPR control. Ali et al. presented a comprehensive survey of beamforming techniques for massive MIMO systems and their relevance to NOMA [15]. The survey discusses interference management, user coupling, and power allocation. The authors emphasized intelligent and adaptive signal processing to handle multiuser interference, indirectly motivating graph-based modeling for NOMA systems. Ahmed et al. reviewed the hybrid beamforming architectures for 5G systems [16]. The survey analyzed the system models, complexity, and energy efficiency issues. The authors highlighted the increasing role of adaptive and intelligent algorithms in multiuser signal optimization, supporting the relevance of structure-aware physical layer designs. Belkacem et al. analyzed the performance of NOMA systems under high-power amplifier nonlinearity [17]. The analytical and simulation results showed a severe BER degradation when the PAPR was high. This study confirms that PAPR reduction is critical for preserving SIC performance and achieving reliable NOMA transmission. Hilario-Tacuri et al. investigated the BER performance of NOMA-OFDM systems with nonlinear HPAs with memory effects [18]. The results revealed that nonlinear distortion disproportionately affects low-power users. This study emphasizes the need for fairness-aware and selective PAPR reduction techniques to maintain the NOMA performance. Ding et al. studied the impact of residual hardware impairments on NOMA-based amplify-and-forward relay networks [19]. The analytical results show the performance floors caused by nonlinear distortions and hardware imperfections. The authors concluded that waveform conditioning techniques such as PAPR reduction are essential for mitigating hardware-induced degradation. Tan et al. proposed a modified PTS-based PAPR reduction technique for ACO-OFDM in visible-light communication systems [20]. The scheme achieves PAPR reduction while preserving the optical signal constraints. Simulation results show an improved BER and reduced peak power, demonstrating the effectiveness of structure-aware selective techniques. Recent surveys of graph representation learning for wireless networks have demonstrated how graph models capture interference, connectivity, and user relationships. These studies applied graph learning to clustering, resource allocation, and scheduling. These studies provide a strong motivation for graph-guided signal processing and suggest that graph metrics can effectively guide adaptive companding for PAPR reduction in NOMA systems.

The authors in [21] propose a power-coefficient-aware adaptive companding scheme for PAPR reduction in downlink PD-NOMA-OFDM systems. The method intelligently adjusts companding based on user power levels, improving BER and PAPR performance. While effective, it primarily relies on amplitude-domain adaptation and does not explicitly capture inter-user interference structure or signal coupling. In contrast, the proposed GGAC framework exploits graph-based signal representation to model multiuser coupling, enabling more effective and selective companding for peak suppression.

More broadly, recent studies highlight the role of advanced computational frameworks and energy-efficient design in next-generation networks. Yang et al. [22] illustrate how computational intelligence can enhance information processing in complex data-driven systems, while Saxena et al. [23] emphasize the importance of low-energy signal-processing strategies for sustainable telecom infrastructures. These perspectives align with the motivation of the present work, in which the proposed GGAC framework leverages structure-aware computational modeling to reduce PAPR—and thereby improve power-amplifier efficiency and energy consumption—in PD-NOMA systems.

## Background and basic terminology

Power-Domain Non-Orthogonal Multiple Access (PD-NOMA) is an advanced multiple access technique designed to enhance spectral efficiency, connectivity, and fairness in modern wireless communication systems, such as 5G and beyond-5G networks. Unlike conventional orthogonal multiple access (OMA) schemes, in which users are allocated distinct time, frequency, or code resources, PD-NOMA allows multiple users to share the same time–frequency resource block simultaneously. The key principle of PD-NOMA is superposition coding at the transmitter and SIC at the receiver, which together enable efficient multiuser signal separation [24].

**Fig 1:**
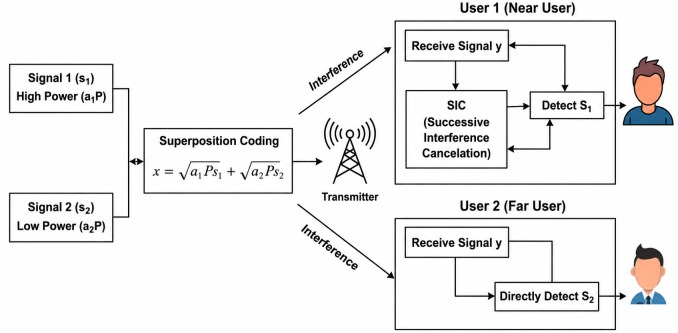
Schematic of PD-NOMA waveform.

In PD-NOMA, the data symbols of multiple users are superimposed in the power domain, with different power levels assigned based on the users’ channel conditions. Typically, users with weaker channel gains (such as cell-edge users) are allocated a higher transmission power, whereas users with stronger channel gains receive lower power. This power allocation strategy improves user fairness and ensures reliable reception for all users. At the receiver side, SIC is employed to decode the superimposed signals. The receiver first decodes the strongest signal (usually the one with the highest power), subtracts it from the received composite signal, and then successively decodes the remaining user signals. PD-NOMA offers several advantages over OMA. It significantly improves the spectral efficiency by serving multiple users on the same resource block, making it well suited for dense networks and massive connectivity scenarios. It also supports diverse quality-of-service requirements, which are essential for applications such as enhanced mobile broadband, massive IoT, and ultra-reliable low-latency communications. Furthermore, PD-NOMA is compatible with key 5G technologies, such as OFDM, massive MIMO, cooperative relaying, and millimeter-wave communications [25]. However, PD-NOMA introduces new challenges, including increased receiver complexity owing to SIC, sensitivity to channel estimation errors, and higher PAPR when combined with multicarrier modulation. Addressing these challenges is crucial for practical realization of PD-NOMA systems in next-generation wireless networks. Fig 1 shows a schematic of the PD-NOMA waveform.

In a power-domain NOMA (PD-NOMA) system, multiple user signals are superimposed in the power domain and transmitted simultaneously over the same time–frequency resource. Let us consider a downlink PD-NOMA system with *K* users sharing the same multicarrier waveform. Each user’s modulated baseband signal is assigned a distinct power coefficient according to its channel condition. A composite NOMA transmit signal is formed by superposition coding, which inherently increases the dynamic range of the transmitted signal and leads to a high PAPR.

Let *x*_*k*_(*t*) denote the normalized baseband signal of the *k*-th user, and let *P*_*k*_ represent the power allocation coefficient for that user, where $\sum_{k=1}^{K}P_{k}=1$. The superimposed PD-NOMA transmit signal *s*(*t*) can be expressed as [26]


$$\begin{array}{c} s(t)=\sum_{k=1}^{K}\sqrt{P_{k}}\;x_{k}(t) \end{array}$$ (1)

When PD-NOMA is combined with multicarrier modulation such as OFDM, the discrete-time baseband signal after the inverse fast Fourier transform (IFFT) over *N* subcarriers is given by


$$\begin{array}{c} s[n]=\frac{1}{\sqrt{N}}\sum_{m=0}^{N-1}\left(\sum_{k=1}^{K}\sqrt{P_{k}}\;X_{k}[m]\right)e^{j\frac{2\pi mn}{N}},\;n=0,1,\ldots ,N-1 \end{array}$$ (2)

where $X_{k}[m]$ represents the modulated symbol of the *k*-th user on the *m*-th subcarrier. Owing to the coherent addition of multiple user signals and subcarriers, large instantaneous peaks may occur in *s*[*n*].

The Peak-to-Average Power Ratio (PAPR) of the PD-NOMA transmit signal is defined as the ratio of the maximum instantaneous power to the average signal power, and is mathematically expressed as


$$\begin{array}{c} PAPR=\frac{\max_{0\leq n<N}|s[n]{|}^{2}}{𝔼\left[|s[n]{|}^{2}\right]} \end{array}$$ (3)

For practical evaluation and comparison, PAPR is often expressed in decibels (dB) as


$$\begin{array}{c} {PAPR}_{dB}=10\log_{10}\left(\frac{\max_{0\leq n<N}|s[n]{|}^{2}}{𝔼\left[|s[n]{|}^{2}\right]}\right) \end{array}$$ (4)

This formulation shows that in PD-NOMA systems, the superposition of multiple users with unequal power levels significantly increases the peak signal power, while the average power remains bounded, resulting in a higher PAPR compared to conventional single-user or orthogonal multiple access systems. Consequently, effective PAPR reduction techniques are essential to ensure the efficient and distortion-free operation of power amplifiers in PD-NOMA-based wireless transmitters.

### CCDF formulation of PAPR in power-domain NOMA systems

While the PAPR of a single transmit block provides limited insight, the system performance is typically evaluated statistically using the Complementary Cumulative Distribution Function (CCDF) of the PAPR. CCDF represents the probability that the PAPR of a transmitted signal exceeds a given threshold and is widely used to compare PAPR reduction techniques. Let PAPR(*s*) denote the PAPR of the PD-NOMA transmit signal *s*[*n*]. The CCDF of the PAPR is defined as the probability that the PAPR is greater than a predefined threshold γ, and is expressed as follows:


$$\begin{array}{c} CCDF(\gamma )=\mathrm{Pr}\left(PAPR(s)>\gamma \right) \end{array}$$ (5)

Substituting the PAPR definition, the CCDF can be written as:


$$\begin{array}{c} CCDF(\gamma )=\mathrm{Pr}\left(\frac{\max_{0\leq n<N}|s[n]{|}^{2}}{𝔼\left[|s[n]{|}^{2}\right]}>\gamma \right) \end{array}$$ (6)

In practical simulations, the CCDF is obtained empirically by generating a large number of PD-NOMA symbols and computing the fraction of symbols whose PAPR exceeds threshold γ. A lower CCDF curve indicates better PAPR performance, which directly translates to reduced power amplifier back-off and improved energy efficiency. These formulations justify the need for selective and structure-aware PAPR reduction techniques, such as Graph-Guided Adaptive Companding, which can exploit user coupling, channel gains, and signal structures to suppress dominant peaks while preserving SIC performance in both downlink and uplink PD-NOMA systems.

## Proposed graph-guided adaptive companding (GGAC)

**Fig 2:**
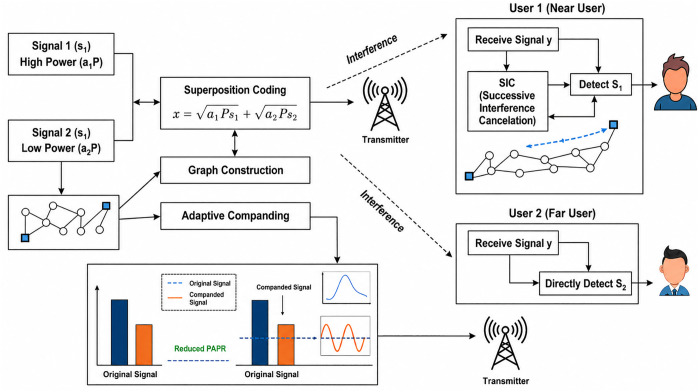
Proposed system model.

Graph-Guided Adaptive Companding (GGAC) is an intelligent and structure-aware PAPR reduction technique specifically designed for power-domain NOMA (PD-NOMA) systems, where multiple users share the same time–frequency resources through superposition coding. In conventional PAPR reduction methods such as clipping, fixed μ-law companding, selective mapping, and partial transmit sequence techniques, the nonlinear transformation is applied uniformly or based on global signal statistics, without considering the inherent multiuser coupling, unequal power allocation, and SIC sensitivity of PD-NOMA signals. Even existing adaptive companding approaches typically rely only on instantaneous amplitude or average power, which can lead to over-compression of low-power users and degradation of SIC performance. To overcome these limitations, GGAC introduces a graph-based signal representation that explicitly models the relationships among signal components, users, and subcarriers. In the proposed GGAC framework, the PD-NOMA transmit signal is mapped onto a graph where nodes represent signal samples, subcarriers, or user components, and edges capture interference coupling, power similarity, or correlation. Graph metrics such as node degree, weighted strength, or local energy are extracted to identify peak-dominant and SIC-critical components. Based on these metrics, companding parameters are adaptively assigned in a non-uniform manner, enabling stronger compression for high-impact components and mild or negligible compression for low-impact or SIC-sensitive components. This selective adaptation allows GGAC to effectively suppress signal peaks while minimizing nonlinear distortion. The novelty of the proposed GGAC lies in its waveform-level application of graph intelligence, which fundamentally differentiates it from existing graph-guided methods that are primarily used for user clustering, power allocation, or scheduling at higher layers. Unlike conventional graph-guided adaptive schemes, the proposed method directly controls nonlinear signal shaping using graph-derived importance measures, without requiring side information, iterative optimization, or learning-based training. As a result, the proposed GGAC achieves a superior trade-off between PAPR reduction, BER performance, and SIC robustness, making it a novel, low-complexity, and practically viable solution for 5G and beyond-5G PD-NOMA systems. The block-level implementation of the proposed step-wise GGAC framework is illustrated in Fig 2. The figure depicts the complete transmitter–receiver chain, including PD-NOMA signal formation, graph construction, importance metric computation, adaptive parameter assignment, nonlinear companding, channel transmission, inverse companding, and SIC-based multi-user detection.

In a power-domain NOMA (PD-NOMA) system, a high PAPR arises owing to the superposition of multicarrier signals of multiple users with unequal power levels. The proposed Graph-Guided Adaptive Companding (GGAC) reduces the PAPR by introducing node-wise graph-driven nonlinear compression instead of applying a uniform or globally adaptive companding function. Let the discrete-time downlink PD-NOMA transmit signal be expressed as


$$\begin{array}{c} s[n]=\sum_{k=1}^{K}\sqrt{P_{k}}\;x_{k}[n],\;n=0,1,\ldots ,N-1 \end{array}$$ (7)

where *x*_*k*_[*n*] is the normalized multicarrier signal of the *k*-th user and *P*_*k*_ is the power allocation coefficient. Signal *s*[*n*] is mapped onto a graph. The graph construction and metric computation are performed on the original, un-companded transmit signal *s*[*n*] prior to the application of adaptive companding.


$$\begin{array}{c} 𝒢=(𝒱,ℰ,𝒲) \end{array}$$ (8)

where each node $v_{i}\in 𝒱$ corresponds to a signal sample *s*[*i*]. The weighted adjacency matrix 𝒲 is defined as


$$\begin{array}{c} W_{ij}=f(|s[i]|,|s[j]|,\rho_{ij}) \end{array}$$ (9)

where $\rho_{ij}$ denotes the correlation or interference coupling between samples *i* and *j*. Here, the function f(·) is explicitly defined through the exponential amplitude-similarity kernel combined with the normalized correlation term. The coupling term *c*_*ij*_ is represented by the normalized correlation $\rho_{ij}$, which captures phase-aligned interference coupling between signal samples in a scale-invariant manner.

The correlation term $\rho_{ij}$ is computed using a normalized inner product, which captures phase-aligned similarity between signal samples while ensuring scale invariance. The use of an exponential amplitude-similarity kernel combined with this correlation metric enables accurate modeling of both power proximity and interference coupling in the constructed graph. This explicit formulation ensures that the graph construction process is fully deterministic and reproducible. The composite PD-NOMA transmit signal *s*[*n*] is represented as a weighted graph.


$$\begin{array}{c} 𝒢=(𝒱,ℰ,𝐖) \end{array}$$ (10)

where each node $v_{i}\in 𝒱$ corresponds to a time-domain signal sample *s*[*i*], and edges represent the relationship between signal components. To ensure computational efficiency and locality, edges are constructed only within a finite neighborhood defined as:


$$\begin{array}{c} 𝒩(i)=\{j||i-j|\leq K_{g}\} \end{array}$$ (11)

where *K*_*g*_ is the neighborhood size controlling graph sparsity. The weighted adjacency matrix 𝒲 is defined as


$$\begin{array}{c} W_{ij}=\left\{ \begin{array}{cc} \frac{\exp\left(-\frac{\left||s[i]|-|s[j]|\right|}{\sigma }\right)\cdot \rho_{ij}}{\;}, & j\in 𝒩(i)\\ 0, & \text{otherwise} \end{array}\right. \end{array}$$ (12)

where exp(·) captures amplitude similarity, giving higher weights to samples with similar magnitudes, σ is a scaling factor controlling sensitivity, and $\rho_{ij}$ represents the normalized correlation between samples given by:


$$\begin{array}{c} \rho_{ij}=\frac{s[i]\;s[j{]}^{*}}{\left|s[i]|\;|s[j]\right|} \end{array}$$ (13)

The parameters governing graph construction are explicitly defined to ensure reproducibility and clarity. The neighborhood size *K*_*g*_ determines the number of adjacent samples considered for edge formation and is fixed to *K*_*g*_ = 4 in this work, which provides a balance between capturing local signal interactions and maintaining computational efficiency. The kernel scaling parameter σ controls the sensitivity of the amplitude similarity function. A moderate value of σ=0.5 (normalized scale) is selected to ensure that samples with small amplitude differences have strong connectivity, while distant samples are weakly connected. The node importance metric $\Gamma_{i}$ is normalized using a max-based normalization scheme:


$$\begin{array}{c} {\tilde{\Gamma }}_{i}=\frac{\Gamma_{i}}{\max(\Gamma )}, \end{array}$$ (14)

ensuring that all values lie within the range [0, 1], thereby enabling stable and bounded mapping of companding parameters.

A graph importance metric for node *i* is computed as


$$\begin{array}{c} \Gamma_{i}=\sum_{j\in 𝒩(i)}W_{ij} \end{array}$$ (15)

which captures the contribution of the node to peak formation and interference coupling.

Unlike conventional companding, which employs a fixed or globally adaptive parameter, the proposed GGAC assigns node-specific companding parameters derived from graph-structural metrics. A higher value of $\Gamma_{i}$ indicates that the corresponding signal sample contributes more significantly to peak formation and inter-carrier interference coupling.

The graph Laplacian ℒ quantifies signal smoothness over the constructed graph, where larger values of ℰ(𝐬) reflect stronger local variations and a greater tendency toward peak-dominant behavior. The graph Laplacian is defined as:


$$\begin{array}{c} ℒ=𝒟-𝒲, \end{array}$$ (16)

where 𝒲 is the weighted adjacency matrix, and 𝒟 is the diagonal degree matrix with entries $D_{ii}=\sum_{j}W_{ij}$.

To further characterize the structural properties of the constructed signal graph, we introduce the graph Laplacian matrix as a fundamental operator for capturing signal variation across the graph. The Laplacian encodes both the connectivity and the interaction strength among signal components through the weighted adjacency matrix. Leveraging this representation, it becomes possible to analyze how local differences between interconnected nodes contribute to the overall signal behavior. In particular, large variations between strongly connected nodes are indicative of peak-dominant components and localized irregularities in the composite waveform. This perspective establishes a theoretical basis for relating graph topology to signal characteristics, thereby supporting the use of graph-derived importance metrics within the proposed companding framework.

The total variation of the signal **s** over the graph is expressed as the graph Dirichlet energy:


$$\begin{array}{c} ℰ(𝐬)=\int^{⊤}𝐋\;𝐬=\sum_{i,j}W_{ij}(s[i]-s[j]{)}^{2}. \end{array}$$ (17)

Although the proposed GGAC framework does not explicitly perform spectral graph filtering, the node importance metric $\Gamma_{i}$ in (15) can be interpreted as a localized approximation of graph-induced signal variation. This establishes a principled connection between the proposed structure-aware companding strategy and classical graph signal processing theory, while preserving low computational complexity.

The normalization of the adaptive companding parameter ensures that each $\alpha_{i}$ lies within the predefined range $\left[\alpha_{\min},\alpha_{\max}\right]$, thereby maintaining controlled and stable nonlinear transformation across all signal components. In this framework, the companding strength is directly governed by the graph importance metric $\Gamma_{i}$. Specifically, nodes with higher $\Gamma_{i}$, which correspond to peak-dominant or highly coupled signal components, are assigned larger values of $\alpha_{i}$, resulting in stronger compression to effectively suppress high-amplitude peaks. Conversely, nodes with lower $\Gamma_{i}$, typically associated with low-power or less significant components, are assigned smaller $\alpha_{i}$, ensuring minimal distortion and preserving the integrity of SIC-sensitive signals. To enable node-specific nonlinear compression, the companding parameter $\alpha_{i}$ is adaptively assigned based on normalized graph importance:


$$\begin{array}{c} \alpha_{i}=\alpha_{\min}+(\alpha_{\max}-\alpha_{\min})\cdot \frac{\Gamma_{i}}{\max_{k}\Gamma_{k}} \end{array}$$ (18)

The normalization ensures that $\alpha_{i}$ lies within $\left[\alpha_{\min},\alpha_{\max}\right]$, providing controlled nonlinear processing. Higher $\Gamma_{i}$ leads to stronger compression, while lower $\Gamma_{i}$ results in minimal distortion. This normalization ensures that the companding parameters remain bounded and stable, while enabling stronger compression for peak-dominant samples and minimal distortion for low-power components. The stability of the proposed graph-guided mechanism under varying channel conditions is ensured by the fact that graph construction and weight computation are performed on the transmit signal prior to channel distortion. Consequently, the graph structure depends only on the inherent signal characteristics, such as power distribution and interference coupling, and not on instantaneous channel realizations. Furthermore, the adaptive companding parameter is normalized within a bounded range $\left[\alpha_{\min},\;\alpha_{\max}\right]$, ensuring controlled nonlinear transformation and preventing instability. The robustness of this design is further validated through simulations under imperfect channel estimation and residual interference conditions, where consistent performance gains are observed. This suppresses peaks while preserving SIC-sensitive low-power users. The companded signal is obtained as:


$$\begin{array}{c} y[i]=C_{\alpha_{i}}(s[i]) \end{array}$$ (19)

For μ-law companding, the nonlinear function is defined as:


$$\begin{array}{c} C_{\alpha_{i}}(s[i])=\frac{\ln(1+\alpha_{i}|s[i]|)}{\ln(1+\alpha_{i})}\cdot sgn(s[i]) \end{array}$$ (20)

where sgn(·) denotes the sign function. Unlike conventional companding schemes that employ a single global parameter, the proposed approach introduces a graph-driven, node-specific nonlinear transformation, where the compression strength is directly proportional to the structural importance of each signal component.

The proposed formulation provides a fully deterministic and reproducible mapping from the PD-NOMA signal to graph space, followed by a normalized parameter adaptation and nonlinear transformation. Each step—from graph construction to companding—is mathematically defined, ensuring that the proposed GGAC framework can be directly implemented without ambiguity.

### Explicit weight function explanation

The graph construction in the proposed GGAC framework is fully deterministic and is based on the composite transmit signal prior to companding. Each time-domain sample is represented as a node, and edges are formed within a finite neighborhood to ensure sparsity and computational efficiency. The edge weights are computed using a joint amplitude–correlation model given by:


$$\begin{array}{c} W_{ij}=\exp\;\left(-\frac{\left|\;|s[i]|-|s[j]|\;\right|}{\sigma }\right)\cdot \rho_{ij},\;j\in 𝒩(i), \end{array}$$ (21)

where the exponential kernel captures amplitude similarity, assigning higher weights to samples with similar magnitudes, while the normalized correlation term is defined as


$$\begin{array}{c} \rho_{ij}=\frac{s[i]\;s^{*}[j]}{\left|s[i]|\;|s[j]\right|}. \end{array}$$ (22)

The proposed model captures phase-aligned interference coupling. This formulation ensures that both power proximity and interference relationships are explicitly captured. To limit computational complexity and focus on local peak interactions, edges are constructed only within a predefined neighborhood $𝒩(i)=\{j:|i-j|\leq K_{g}\}$. This results in a sparse graph structure with complexity $𝒪(NK_{g})$, making the approach scalable for large subcarrier sizes.

### Inverse companding and SIC-aware receiver model

To ensure clarity and reproducibility, the proposed GGAC framework is implemented as a deterministic sequence of operations. The method accepts as inputs the user signals *x*_*k*_[*n*], the power allocation coefficients *P*_*k*_, and the companding bounds $\alpha_{\min}$ and $\alpha_{\max}$.

The procedure unfolds as follows. First, the composite PD-NOMA signal is constructed via superposition coding. Next, the time-domain signal is mapped onto a sparse graph, in which each sample is treated as a node and edges are established based on local amplitude similarity and temporal correlation within a predefined neighborhood window. A graph-based node importance metric is then computed for each node to identify peak-dominant and strongly coupled signal components. From this metric, node-specific companding parameters are assigned through a normalized linear mapping onto the interval $\left[\alpha_{\min},\;\alpha_{\max}\right]$. These parameters are subsequently applied to perform nonlinear companding on each signal sample, yielding a transmit signal with significantly reduced PAPR. At the receiver, the identical deterministic graph construction and parameter mapping are reconstructed to execute inverse companding, followed by successive interference cancellation (SIC) for multi-user signal recovery. The complete step-by-step procedure is summarized in Algorithm 1.

**Algorithm 1 Graph-Based Adaptive Companding (GGAC) for PD-NOMA**

**Input:**

• Number of users *K*

• User signals *x*_*k*_[*n*]

• Power allocation coefficients *P*_*k*_

• Companding bounds $\alpha_{\min},\;\alpha_{\max}$

• Neighborhood size *K*_*g*_

**Output:**

• Low-PAPR companded signal *y*[*n*]

**Steps:**

1:  Generate multicarrier signals *x*_*k*_[*n*] for all users k=1,…,K

2:  Form the composite PD-NOMA signal via superposition coding:

$s[n]=\sum_{k=1}^{K}\sqrt{P_{k}}\;x_{k}[n]$

3:  Construct the signal graph *G* = (*V*, *E*, **W**):

∘ Treat each sample *s*[*i*] as a node $v_{i}\in V$

∘ Connect nodes within a local neighborhood of size *K*_*g*_

4:  Compute edge weights *W*_*ij*_ based on amplitude similarity and temporal correlation between nodes *v*_*i*_ and *v*_*j*_

5:  Compute the node importance metric for each node:

$\Gamma_{i}=\sum_{j\in 𝒩(i)}W_{ij}$

6:  Assign the node-specific adaptive companding parameter:

$\alpha_{i}=\alpha_{\min}+\left(\alpha_{\max}-\alpha_{\min}\right)\frac{\Gamma_{i}}{\max(\Gamma )}$

7:  Apply nonlinear companding to each signal sample:

$y[i]={𝒞}_{\alpha_{i}}\;\left(s[i]\right)$

8:  Transmit the companded signal *y*[*n*]

9:  **At the receiver:**

∘ Reconstruct the signal graph using the same deterministic procedure

∘ Recompute node-specific parameters $\alpha_{i}$

∘ Apply inverse companding: $\hat{s}[i]={𝒞}_{\alpha_{i}}^{-1}(y[i])$

∘ Perform successive interference cancellation (SIC) for multi-user signal recovery

At the receiver side of a PD-NOMA system employing the proposed GGAC, the received signal undergoes inverse companding, followed by successive interference cancellation (SIC). Because the companding parameters are deterministically derived from the graph-based rules known at both the transmitter and receiver, no explicit side information is required. Let the received downlink PD-NOMA signal be expressed as:


$$\begin{array}{c} r[n]=h\;y[n]+w[n] \end{array}$$ (23)

where *y*[*n*] is the graph-guided companded transmit signal, *h* is the channel coefficient, and *w*[*n*] denotes additive white Gaussian noise. For each signal component *y*[*i*], inverse companding is applied using the same node-specific parameter $\alpha_{i}$ derived from the graph metrics. The reconstruction of node-specific companding parameters at the receiver is achieved using the same deterministic graph construction and parameter mapping rules applied at the transmitter. Specifically, the received signal samples are first used to reconstruct the graph 𝒢 using the predefined neighborhood size and weight function. The graph importance metric $\Gamma_{i}$ is then recomputed, followed by normalization to obtain the corresponding companding parameters $\alpha_{i}$. Because this process depends only on known system parameters and the received signal, no explicit side information is required. This ensures practical implementability and preserves synchronization between transmitter and receiver operations. The decompanded signal is obtained as follows:


$$\begin{array}{c} \hat{s}[i]=C_{\alpha_{i}}^{-1}(y[i]) \end{array}$$ (24)

For logarithmic companding, the inverse function is given by


$$\begin{array}{c} \hat{s}[i]=sgn(y[i])\left(\frac{e^{\alpha_{i}|y[i]|}-1}{\alpha_{i}}\right) \end{array}$$ (25)

This selective inverse companding restores the original signal amplitude while limiting the distortion introduced during the peak compression. After inverse companding, SIC is applied to decode the superimposed user signals. Assuming users are ordered according to descending received power $P_{1}|h_{1}{|}^{2}\geq P_{2}|h_{2}{|}^{2}\geq \cdots \geq P_{K}|h_{K}{|}^{2}$, the reconstructed signal can be written as


$$\begin{array}{c} \hat{s}[n]=\sum_{k=1}^{K}\sqrt{P_{k}}\;h_{k}\;x_{k}[n]+\tilde{w}[n] \end{array}$$ (26)

At the *k*-th SIC stage, the signal of user *k* is detected as:


$$\begin{array}{c} {\hat{x}}_{k}[n]=𝒟\left(\hat{s}[n]-\sum_{j=1}^{k-1}\sqrt{P_{j}}\;h_{j}\;{\hat{x}}_{j}[n]\right) \end{array}$$ (27)

where 𝒟(·) denotes the symbol-decision operator. The uniqueness of the proposed GGAC lies in the fact that node-specific companding parameters preserve the power-domain hierarchy, that is,


$$\begin{array}{c} \alpha_{i}\propto \Gamma_{i}\Rightarrow \text{Dominant components are compressed more strongly} \end{array}$$ (28)

This adaptive mapping ensures that the effective distortion introduced by companding remains proportional to the signal dominance, i.e., $\sigma_{d}^{2}(\alpha_{1})>\sigma_{d}^{2}(\alpha_{2})>\cdots >\sigma_{d}^{2}(\alpha_{K})$, thereby preserving the power-domain ordering required for reliable SIC. Consequently, the proposed method mitigates error propagation across successive decoding stages by limiting distortion on low-power users, which are most vulnerable to SIC failure.

Unlike conventional uniform companding, this ensures that low-power users are not overcompressed, thereby reducing SIC error propagation. Consequently, the proposed GGAC improves both PAPR reduction and SIC reliability, achieving a balanced trade-off between nonlinear distortion suppression and multiuser detection accuracy. In PD-NOMA systems employing GGAC, node-specific companding introduces a controlled nonlinear distortion that varies across the signal components. After inverse companding, the residual distortion can be modeled as an equivalent additive distortion noise, whose variance depends on the graph-adaptive parameter $\alpha_{i}$. This allows the SINR to be analytically characterized while preserving tractability. After inverse companding and channel equalization, the reconstructed signal can be written as:


$$\begin{array}{c} \hat{s}[n]=\sum_{k=1}^{K}\sqrt{P_{k}}\;h_{k}\;x_{k}[n]+d[n]+w[n] \end{array}$$ (29)

where *d*[*n*] denotes the residual companding distortion, and *w*[*n*] is the channel noise. At the *k*-th SIC stage, the instantaneous SINR for user *k* is expressed as follows: The signal-to-interference-plus-noise ratio (SINR) for user *k* is expressed as:


$${\text{SINR}}_{k}=\frac{P_{k}{\left|h_{k}\right|}^{2}}{\begin{array}{l} \underset{\text{same-frequency interference}}{\underbrace{\sum_{i>k}P_{i}{\left|h_{k}\right|}^{2}}}+\underset{\text{adjacent channel interference}}{\underbrace{I_{\text{adj}}}}\\ +\underset{\text{residual SIC interference}}{\underbrace{\sum_{j<k}\varepsilon_{j}P_{j}{\left|h_{k}\right|}^{2}}}+\underset{\text{GGAC distortion}}{\underbrace{\sigma_{d}^{2}(\alpha_{k})}}+\underset{\text{noise}}{\underbrace{\sigma_{w}^{2}}} \end{array}}$$ (30)

where $\sum_{i>k}P_{i}|h_{k}{|}^{2}$ represents same-frequency interference from higher-power users, *I*_adj_ denotes adjacent channel interference, and $\sum_{j<k}\epsilon_{j}P_{j}|h_{k}{|}^{2}$ models residual interference arising from imperfect successive interference cancellation (SIC), with $\epsilon_{j}\in [0,1]$ denoting the error propagation factor at the *j*-th SIC stage. The term $\sigma_{d}^{2}(\alpha_{k})$ represents the nonlinear distortion variance introduced by the GGAC companding operation, and $\sigma_{w}^{2}$ is the additive white noise variance.

The residual interference factor $\epsilon_{j}\in [0,1]$ quantifies the fraction of interference power that remains after imperfect SIC, thereby capturing error propagation across successive decoding stages. Larger values of $\epsilon_{j}$ indicate stronger residual interference from previously decoded users. This formulation is widely adopted in the NOMA literature to model SIC imperfections, treating residual interference as a scaled version of the incompletely canceled signal. It is commonly employed in both analytical and simulation-based studies for SINR and outage probability evaluation. Furthermore, imperfect SIC has been shown to introduce non-negligible residual interference that significantly degrades system performance, thereby justifying the inclusion of an explicit error propagation factor in the SINR expression [27]. Under ideal SIC, $\varepsilon_{k}=0$ and the expression reduces to the standard SINR formulation. Unlike conventional companding, where $\sigma_{d}^{2}$ is constant for all users, GGAC yields


$$\begin{array}{c} \sigma_{d}^{2}(\alpha_{k})=g(\alpha_{k}),\;\alpha_{k}\propto \Gamma_{k} \end{array}$$ (31)

Eq (31) indicates that dominant users experience stronger compression but controlled distortion, whereas weaker users experience minimal distortion, preserving the SIC order. Using the derived SINR, the BER for user *k* under the GGAC can be approximated based on the modulation scheme. For example, for QPSK modulation, the BER is given by:


$$\begin{array}{c} {BER}_{k}^{GGAC}\approx Q\left(\sqrt{2\;{SINR}_{k}^{GGAC}}\right) \end{array}$$ (32)

where Q(·) is the Gaussian Q-function. For M-QAM modulation, the BER expression can be approximated as:


$$\begin{array}{c} {BER}_{k}^{GGAC}\approx \frac{4}{\log_{2}M}\left(1-\frac{1}{\sqrt{M}}\right)Q\left(\sqrt{\frac{3\;{SINR}_{k}^{GGAC}}{M-1}}\right) \end{array}$$ (33)

In conventional companding, a single global parameter α leads to:


$$\begin{array}{c} \sigma_{d}^{2}(\alpha_{k})=\sigma_{d}^{2}\;\forall k \end{array}$$ (34)

Eq (34) disproportionately degrades low-power users and increases SIC error propagation. In contrast, GGAC ensures


$$\begin{array}{c} \sigma_{d}^{2}(\alpha_{1})>\sigma_{d}^{2}(\alpha_{2})>\cdots >\sigma_{d}^{2}(\alpha_{K}) \end{array}$$ (35)

Eq (35) aligns the distortion with the user dominance. This graph-driven distortion shaping improves the SINR for weaker users and leads to a lower BER across all SIC stages while simultaneously achieving effective PAPR reduction. The proposed Graph-Guided Adaptive Companding (GGAC) reduces the PAPR by selectively suppressing only the signal components responsible for large-amplitude peaks in a power-domain NOMA waveform. First, the transmitted NOMA signal is modeled as a graph, where nodes represent signal samples or user components, and edges capture the power and interference relationships. Graph metrics identify the peak-dominant and highly coupled components that contribute the most to instantaneous power surges. Adaptive companding parameters are then assigned to these components by applying stronger compression to high-impact nodes and minimal compression to low-impact nodes. This targeted, nonuniform compression lowers the peak power while preserving the average power, thereby effectively reducing the PAPR without distorting the SIC-sensitive signals.

For implementation clarity, each stage of the GGAC framework corresponds directly to the mathematical formulations presented above. Specifically, Eq (7) defines the PD-NOMA signal generation (Step 2); Eq (10)–(13) govern the graph construction procedure (Step 3); Eq (14) defines the node importance metric (Step 4); Eq (17) defines the adaptive companding parameter assignment (Step 5); and Eq (18)–(19) define the nonlinear companding operation (Step 6). This one-to-one correspondence between the algorithmic steps and their mathematical definitions ensures that the proposed GGAC method can be implemented in a straightforward and fully reproducible manner, free from ambiguity.

This graph-guided adaptation enables effective PAPR reduction, while minimizing signal distortion and preserving the power hierarchy required for reliable SIC. GGAC improves the power amplifier efficiency, enhances user fairness, and maintains BER performance. Owing to its structure-aware and adaptive nature, GGAC is well suited for 5G and beyond-5G systems, where dense connectivity, energy efficiency, and intelligent physical layer processing are essential. The structure of the proposed graph naturally adapts to variations in the number of users and subcarriers. Specifically, the number of nodes in the graph corresponds to the number of time-domain samples, which is directly proportional to the subcarrier size *N*. As *N* increases, the graph becomes larger but remains sparse due to the localized neighborhood constraint. The number of users influences the graph indirectly through the composite signal formation. As more users are superimposed, the signal exhibits increased interference coupling and dynamic range, which is reflected in the edge weights and node importance metrics. Consequently, the graph structure evolves automatically without requiring explicit redesign, enabling scalable operation across different system configurations. This adaptive behavior ensures that the proposed GGAC framework remains effective for varying network densities and multicarrier settings.

### Theoretical relationship between graph structure and peak formation

In multicarrier PD-NOMA systems, high PAPR arises due to the constructive superposition of multiple subcarriers and users, leading to localized high-energy signal components. These peaks correspond to regions of strong signal variation, which can be effectively characterized using graph signal processing. In this framework, the composite signal is represented as a graph, where structural relationships between signal samples are captured through weighted connections. The Graph Laplacian, defined as 𝐋=𝐃−𝐖, where **W** is the weighted adjacency matrix and **D** is the diagonal degree matrix, provides a fundamental operator for analyzing signal variation over the graph. The associated graph smoothness is quantified using the Laplacian energy


$$\begin{array}{c} E_{G}={𝐬}^{⊤}𝐋\;𝐬=\frac{1}{2}\sum_{i,j}W_{ij}(s[i]-s[j]{)}^{2}, \end{array}$$ (36)

which measures the degree of variation between connected nodes. A lower value of *E*_*G*_ indicates a smooth signal, whereas higher values correspond to strong local variations. In PD-NOMA signals, peak formation is inherently linked to abrupt amplitude changes caused by constructive interference among users and subcarriers, resulting in large differences |s[i]−s[j]| between neighboring samples, particularly when weighted by strong coupling coefficients *W*_*ij*_. As a result, peak-dominant regions contribute disproportionately to the graph Laplacian energy, establishing a direct relationship between graph-domain signal variation and peak formation.

Based on this interpretation, the node importance metric


$$\begin{array}{c} \Gamma_{i}=\sum_{j\in 𝒩(i)}W_{ij} \end{array}$$ (37)

serves as a localized approximation of graph-induced signal variation. Nodes with higher $\Gamma_{i}$ correspond to regions of strong coupling and high energy concentration, enabling efficient identification of peak-dominant components without explicitly computing global Laplacian energy. This provides a theoretical foundation for the use of graph-based modeling, where the structure of the signal is leveraged to guide adaptive processing. Unlike conventional companding methods that rely solely on amplitude-based transformations, the graph representation captures both local variation and interaction among signal components, allowing selective nonlinear compression to be applied where it is most needed. Consequently, the proposed approach achieves effective peak suppression while preserving the integrity of low-power and interference-sensitive components, demonstrating that graph-guided companding is fundamentally aligned with the structural characteristics of PD-NOMA signals rather than serving merely as a weighted computation mechanism.

### Nonlinear HPA modeling using rapp model

To evaluate the practical effectiveness of the proposed PAPR reduction scheme under realistic transmission conditions, a nonlinear high-power amplifier (HPA) model is incorporated using the widely adopted Rapp model. The Rapp model characterizes the amplitude distortion behavior of solid-state power amplifiers (SSPA) without introducing phase distortion, making it suitable for multicarrier systems such as PD-NOMA. The output of the HPA is given by:


$$\begin{array}{c} y(t)=\frac{x(t)}{{\left(1+{\left(\frac{\left|x(t)\right|}{A_{sat}}\right)}^{2p}\right)}^{\;\frac{1}{2p}}}, \end{array}$$ (38)

where *x*(*t*) and *y*(*t*) denote the PA input and output signals, respectively, *A*_sat_ is the saturation amplitude, and *p* is the smoothness factor governing the transition between the linear amplification region and the saturation region. A smaller value of *p* produces a gradual, soft saturation characteristic, whereas larger values of *p* cause the model to approach the behavior of an ideal hard limiter.

In this work, the Rapp model is applied after the companding stage to evaluate the impact of PA-induced nonlinear distortion on BER and SINR performance. This configuration enables a rigorous assessment of how effectively the proposed GGAC method mitigates distortion under practical power amplifier constraints.

## Simulation

To ensure reproducibility and transparency, all simulation parameters and implementation details of the proposed GGAC framework are explicitly defined in this section. The simulations were conducted using MATLAB (2025a), and all results were averaged over independent Monte Carlo realizations to ensure statistical reliability. The simulation parameters are shown in Table 1.

**Table 1:** Simulation parameters of the proposed work.

Parameter	Value
Simulation tool	MATLAB (2025a)
Number of users (*K*)	2, 4, 6, and 12
Subcarriers (*N*)	128, 256, 512
Modulation	QPSK, 16-QAM
Power allocation	Descending coefficients (e.g., 0.6, 0.3, 0.1 for *K* = 3; extended for larger *K*)
Channel model	Rayleigh fading + AWGN
Oversampling factor (*L*)	4
Monte Carlo runs	10^4^
Neighborhood size (*K*_*g*_)	4
Companding range	$\alpha_{\min}=0.5$, $\alpha_{\max}=5$
SIC type	Perfect / Imperfect (specified cases)
Channel estimation error	0%, 20%, 30%
Performance metrics	PAPR (CCDF), BER, SINR, PSD

A downlink PD-NOMA system with up to 12 users sharing the same time–frequency resource block was considered. To assess the scalability and robustness of the proposed method under different multicarrier configurations, multiple subcarrier sizes *N* = 128, 256, and 512 were employed, and the corresponding values are explicitly indicated in each figure. Quadrature phase-shift keying (QPSK) and 16-QAM modulation schemes were used to evaluate performance under different modulation orders. The power allocation coefficients follow a descending order (e.g., *P*_1_ = 0.6, *P*_2_ = 0.3, *P*_3_ = 0.1) to satisfy SIC requirements. A Rayleigh fading channel with additive white Gaussian noise (AWGN) was assumed, with perfect channel state information available at the receiver. The signal was oversampled by a factor of *L* = 4 for accurate PAPR estimation. The companding parameters were set to $\alpha_{\min}=0.5$ and $\alpha_{\max}=5$, while graph construction employed a sparse neighborhood with *K*_*g*_ = 4 adjacent nodes per sample. For performance evaluation, the complementary cumulative distribution function (CCDF) of PAPR, BER versus SNR, and SINR were analyzed. Each simulation result was averaged over 10 × 10 independent Monte Carlo realizations, including independent symbol blocks and channel realizations. Additional trials were conducted to verify convergence, and no significant variation in the performance curves was observed, confirming statistical reliability. Furthermore, to ensure a fair and unbiased comparison, all baseline methods included conventional PD-NOMA without PAPR reduction, fixed μ-law companding, SLM, PTS, PTS-PSO, and learning-based approaches (Autoencoder and ML-RNN), representing conventional, optimization-based, and learning-assisted techniques. All methods were implemented under identical system parameters to ensure a fair and consistent performance evaluation.

All benchmark techniques, including μ-law companding, SLM, PTS, PTS-PSO, and learning-based methods, were implemented under identical system parameters, including the same number of subcarriers, modulation schemes, power allocation strategy, channel model, and oversampling factor, to ensure a fair and unbiased performance comparison.

The performance gains achieved by the proposed method are configuration-dependent and are more pronounced in scenarios with structured signal coupling and a moderate number of subcarriers.

To evaluate the proposed GGAC framework under realistic operating conditions, additional simulations were conducted incorporating practical system impairments. Specifically, channel estimation errors of 20% and 30% were introduced to model imperfect channel state information (CSI) at the receiver, and residual interference was included to emulate imperfect successive interference cancellation (SIC). These scenarios reflect practical wireless environments in which ideal assumptions — such as perfect CSI and error-free SIC — are not guaranteed. The corresponding results are presented in Figs. 8–10 and confirm the robustness of the proposed method against non-ideal channel conditions and practical implementation constraints.

### Baseline configuration and fair comparison protocol

To ensure a fair and unbiased performance evaluation, all baseline methods were implemented under identical system conditions, including the same number of users, subcarriers, modulation schemes, power allocation strategy, channel model, and oversampling factor. Furthermore, the parameters of each baseline method were carefully selected based on standard practices in the literature and, where applicable, tuned to achieve near-optimal performance under the considered system setup. Specifically, classical PAPR reduction techniques such as μ-law companding, Selective Mapping (SLM), and Partial Transmit Sequences (PTS) were configured using commonly adopted parameter ranges, and multiple configurations were tested to ensure representative performance. Optimization-based methods such as PTS-PSO were executed with sufficient population size and iteration count to ensure convergence.

For learning-based methods, including Autoencoder and RNN-based models, the network architectures, training epochs, and learning parameters were selected to ensure convergence and stable performance. These models were trained using sufficiently large datasets generated under the same system assumptions as the proposed method. The final reported results correspond to the best-performing configuration for each baseline method within reasonable computational complexity constraints. This ensures that the observed performance gains of the proposed GGAC method are not due to suboptimal baseline configurations, but rather reflect genuine improvements in PAPR reduction, BER, and spectral efficiency. Table 2 summarizes the configuration and tuning strategy adopted for all baseline methods considered in this study to ensure a fair and unbiased performance comparison.

**Table 2:** Baseline methods and parameter configurations.

Method	Key Parameters	Configuration Strategy
μ-law Companding	μ parameter	Tested over range (μ=2−10); best value selected
SLM	Number of phase sequences (*U*)	*U* = 4, 8, 16 tested; best trade-off chosen
PTS	Number of subblocks (*V*), phase factors	V=4−8; optimized phase set
PTS-PSO	Population size, iterations	Tuned for convergence (20–50 particles, 50–100 iterations)
Autoencoder	Layers, neurons, epochs	Trained until convergence with validation
ML (RNN)	Hidden units, learning rate	Tuned using validation dataset
Conventional PD-NOMA	No PAPR reduction	Baseline reference

For conventional nonlinear techniques such as μ-law companding, the compression parameter μ was varied over a standard range (e.g., 2–10), and the best-performing value was selected based on PAPR reduction performance. For probabilistic techniques such as SLM and PTS, key parameters including the number of phase sequences *U* and number of subblocks *V* were systematically varied, and configurations providing the best trade-off between performance and computational complexity were chosen. The optimization-based PTS-PSO method was configured with sufficient population size and iteration count to ensure convergence to a stable solution.

For learning-based approaches, including the Autoencoder and RNN-based models, network architectures (e.g., number of layers, neurons) and training parameters (e.g., learning rate, epochs) were tuned using validation datasets to achieve stable convergence and reliable performance. Finally, the conventional PD-NOMA system without PAPR reduction is included as a reference baseline. Overall, all baseline methods were evaluated under optimized or near-optimized configurations within practical complexity limits, ensuring that the reported performance improvements of the proposed GGAC method are both fair and meaningful.

All baseline methods are evaluated using their optimized or near-optimized configurations to ensure a fair comparison.

**Fig 3:**
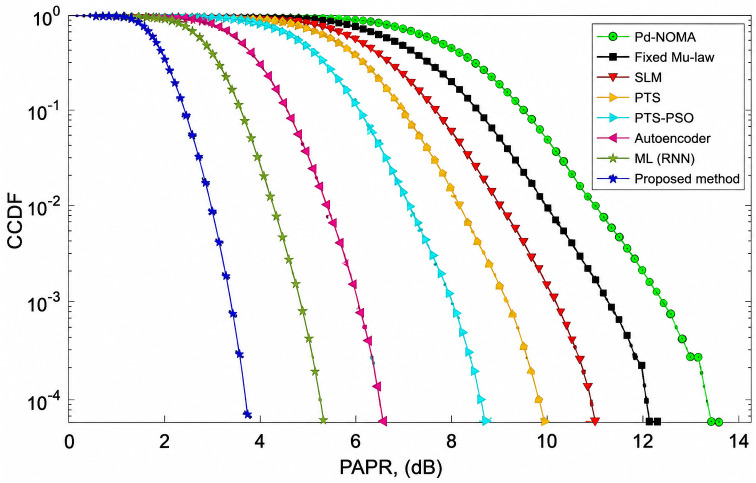
PAPR analysis of PD-NOMA for 512 subcarriers.

Fig 3 illustrates the CCDF-based PAPR performance of the PD-NOMA system employing 512 subcarriers for various PAPR reduction techniques. The CCDF curve represents the probability that the PAPR exceeds a given threshold, where a leftward shift indicates an improved performance. As observed, the conventional PD-NOMA scheme exhibited the highest PAPR, whereas all reduction methods achieved noticeable improvements. At a CCDF level of 10^−3^, the baseline PD-NOMA system attained a PAPR of approximately 12.8 dB. In comparison, fixed μ-law companding, SLM, PTS, and PTS-PSO reduce the PAPR to about 11.6 dB, 10.8 dB, 10.0 dB, and 8.8 dB, respectively. Advanced learning-based approaches further improve the performance, with the Autoencoder and ML (RNN) methods achieving PAPR values of nearly 6.2 dB and 5.2 dB, respectively. The proposed method demonstrated the best performance, achieving a PAPR of approximately 3.9 dB at the same CCDF level. Consequently, the proposed scheme offers a PAPR gain of about 8.9 dB compared to conventional PD-NOMA, while providing gains of 7.7 dB, 6.9 dB, 6.1 dB, and 4.9 dB over fixed μ-law, SLM, PTS, and PTS-PSO techniques, respectively. Moreover, it outperformed the Autoencoder and ML (RNN) approaches by approximately 2.3 dB and 1.3 dB. Unlike learning-based approaches that rely on trained approximations, the proposed method operates directly on instantaneous signal structure, which can provide improved peak suppression under specific configurations without requiring training. These results confirm that the proposed method significantly suppresses high-power peaks, leading to improved power amplifier efficiency and reduced nonlinear distortion, making it highly suitable for PD-NOMA-based systems beyond 5G.

The significant performance gain observed for the proposed method is primarily due to its structure-aware and selective companding mechanism, which suppresses only peak-dominant components while preserving low-power and SIC-sensitive signals. This targeted nonlinear processing reduces distortion more effectively than conventional uniform or optimization-based techniques, leading to consistent improvements across multiple performance metrics.

**Fig 4:**
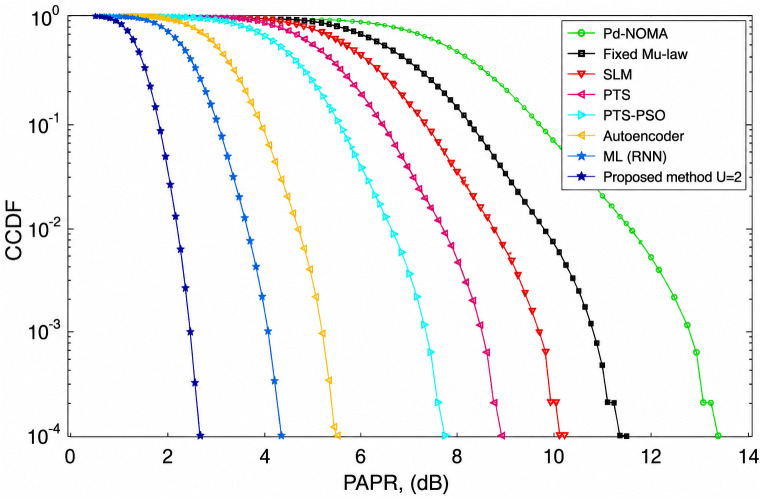
PAPR analysis of PD-NOMA for 256 subcarriers.

Fig 4 shows the CCDF-based PAPR performance of a PD-NOMA system employing 256 subcarriers under different PAPR reduction techniques. As shown in Fig 4, the CCDF indicates the probability that the PAPR exceeds a given threshold, and a leftward shift of the curve signifies a superior PAPR reduction capability. The conventional PD-NOMA scheme shows the worst performance with the highest PAPR, whereas all PAPR reduction methods provide varying degrees of improvement. At a CCDF level of 10^−3^, the baseline PD-NOMA system exhibited a PAPR of approximately 12.6 dB. The fixed μ-law companding technique reduces the PAPR to approximately 10.8 dB, whereas SLM and PTS further improve the performance, achieving PAPR values of nearly 9.8 dB and 8.6 dB, respectively. The optimization-assisted PTS-PSO scheme provides an additional reduction, attaining a PAPR of approximately 7.2 dB at the same CCDF level. Learning-based approaches show enhanced effectiveness, with the autoencoder-based method achieving approximately 5.6 dB, and the ML (RNN) technique further reduces the PAPR to approximately 4.4 dB. The proposed method demonstrates the best performance among all the compared schemes, achieving a significantly lower PAPR of approximately 2.6 dB at CCDF = 10^−3^. Consequently, the proposed approach offers a PAPR gain of about 10.0 dB compared to conventional PD-NOMA, while providing gains of 8.2 dB, 7.2 dB, 6.0 dB, and 4.6 dB over fixed μ-law, SLM, PTS, and PTS-PSO techniques, respectively. Moreover, it outperformed the Autoencoder and ML (RNN) methods by approximately 3.0 dB and 1.8 dB. These results clearly indicate that reducing the number of subcarriers to 256 further enhances the effectiveness of the proposed method, leading to substantial suppression of high-power peaks, improved power amplifier efficiency, and reduced nonlinear distortion in PD-NOMA systems.

**Fig 5:**
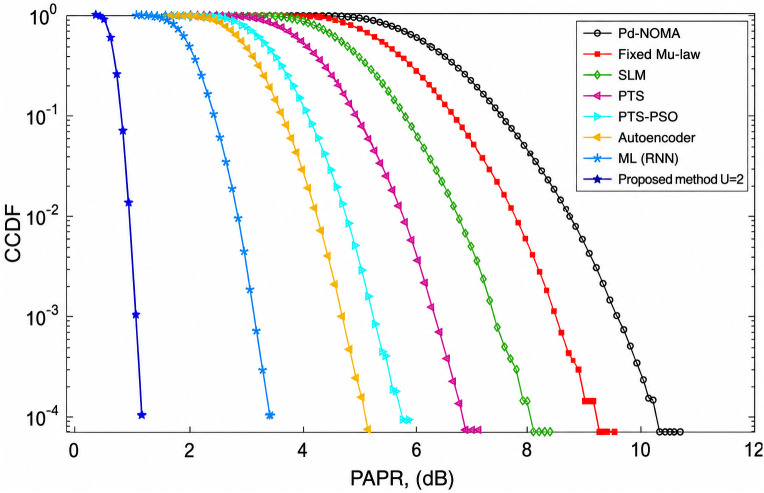
PAPR analysis of PD-NOMA for 128 subcarriers.

Fig 5 shows the CCDF-based PAPR performance of a PD-NOMA system employing 128 subcarriers for various conventional, optimization-based, and learning-assisted PAPR reduction techniques. The CCDF curve reflects the probability that the PAPR exceeds a given threshold, and a noticeable leftward shift of the curve indicates an improved PAPR suppression. Owing to the reduced number of subcarriers, the overall PAPR values are lower than those of the 256- and 512-subcarrier cases; however, clear performance differences among the schemes are still evident. At a CCDF level of 10^−3^, the conventional PD-NOMA system exhibited a PAPR of approximately 9.8 dB, representing the worst-case scenario among the compared methods. Fixed μ-law companding achieves a moderate reduction, yielding a PAPR of around 8.6 dB, while SLM and PTS further reduce the PAPR to approximately 7.4 dB and 6.4 dB, respectively. The PTS-PSO technique provides an additional improvement, attaining a PAPR of nearly 5.6 dB at the same CCDF level. Learning-based approaches demonstrate superior performance, with the autoencoder-based method achieving a PAPR of approximately 4.6 dB, and the ML (RNN) technique further reducing it to approximately 3.6 dB. The proposed method significantly outperforms all other schemes, achieving a notably low PAPR of approximately 1.4 dB at CCDF = 10^−3^. As a result, the proposed approach offers a PAPR gain of about 8.4 dB compared to conventional PD-NOMA and provides gains of 7.2 dB, 6.0 dB, 5.0 dB, and 4.2 dB over fixed μ-law, SLM, PTS, and PTS-PSO techniques, respectively. Furthermore, it outperformed the Autoencoder and ML (RNN) approaches by approximately 3.2 dB and 2.2 dB. These results demonstrate that decreasing the number of subcarriers to 128 substantially enhances the effectiveness of the proposed method, leading to strong suppression of signal peaks, improved power amplifier efficiency, and reduced nonlinear distortion in PD-NOMA systems.

The very low PAPR values observed in this case are attributed to the reduced number of subcarriers (128), where the inherent signal dynamic range is lower compared to larger multicarrier configurations.

The reported performance gains are configuration-dependent and are more pronounced in scenarios with fewer subcarriers and structured signal coupling. To model the impact of practical power amplifier constraints, the Rapp nonlinear HPA model is incorporated into the simulation framework, as described in the subsection above. Dedicated simulation results evaluating BER and SINR under Rapp model distortion represent a direction for future work, alongside hardware nonlinearity analysis using the Saleh AM/AM–AM/PM model.

**Fig 6:**
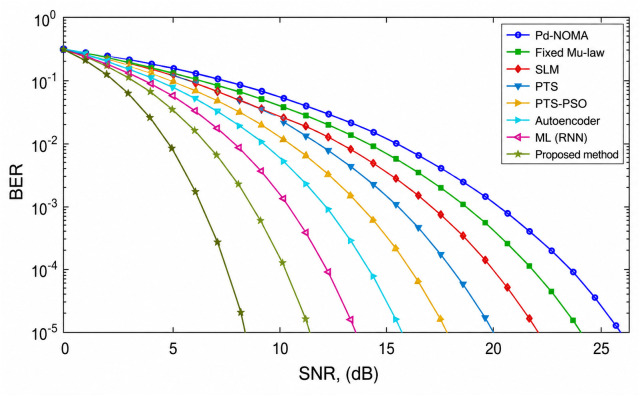
BER analysis of PD-NOMA for 512 subcarriers.

Fig 6 shows the BER performance of a PD-NOMA system with 512 subcarriers under various PAPR reduction techniques as a function of the SNR. The curves indicate that methods achieving stronger PAPR suppression also provide improved BER performance, as reduced PAPR alleviates the nonlinear distortion effects caused by the power amplifier. A clear leftward shift of the BER curve implies that a lower SNR is required to attain the same BER, thereby enhancing system reliability. The conventional PD-NOMA scheme exhibited the poorest performance across the SNR range, whereas the optimization-based and learning-assisted techniques showed noticeable improvements. At a target BER of 10^−3^, the baseline PD-NOMA system required an SNR of approximately 18.5 dB. In comparison, fixed μ-law, SLM, and PTS techniques achieve the same BER at around 16.8 dB, 15.6 dB, and 14.8 dB, respectively, while the PTS-PSO scheme further reduces the required SNR to nearly 13.6 dB. Learning-based approaches provide additional gains, with the Autoencoder and ML (RNN) methods achieving BER = 10^−3^ at approximately 12.4 dB and 11.2 dB, respectively. The proposed method demonstrated the best performance, achieving the target BER at a significantly lower SNR of approximately 9.2 dB. Consequently, the proposed scheme offers an SNR gain of approximately 9.3 dB over conventional PD-NOMA and provides gains of about 7.6 dB, 6.4 dB, 5.6 dB, and 4.4 dB compared with fixed μ-law, SLM, PTS, and PTS-PSO techniques, respectively. Moreover, it outperformed the Autoencoder and ML (RNN) approaches by approximately 3.2 dB and 2.0 dB. These results demonstrate that the proposed method significantly enhances the BER performance by requiring a substantially lower SNR, mainly because of its superior PAPR reduction capability, which effectively mitigates nonlinear distortion in PD-NOMA systems with a large number of subcarriers.

**Fig 7:**
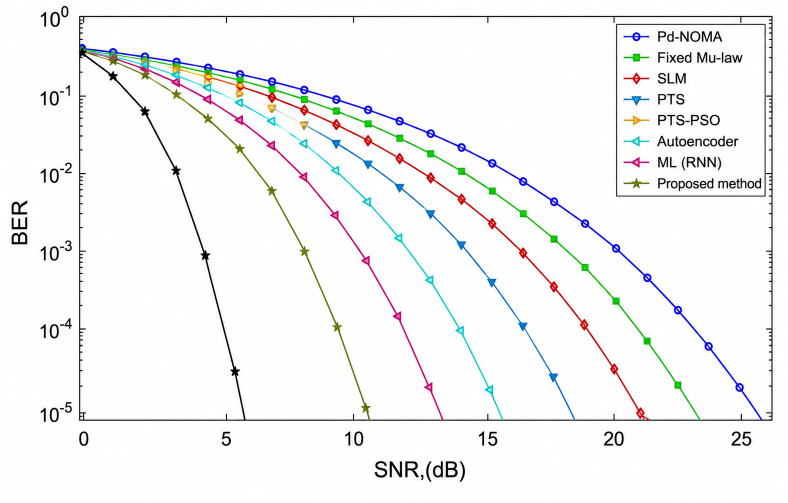
BER analysis of PD-NOMA for 256 subcarriers.

Fig 7 shows the BER performance of a PD-NOMA system with 256 subcarriers using different PAPR reduction techniques as a function of the SNR. It is observed that the conventional PD-NOMA scheme exhibits the poorest BER performance, requiring a significantly higher SNR to achieve reliable transmission owing to the severe nonlinear distortion caused by the high PAPR. Classical techniques such as fixed μ-law companding, SLM, PTS, and PTS-PSO progressively improve the BER performance, whereas learning-based approaches including the autoencoder and ML (RNN) further enhance reliability by better preserving the signal structure. The proposed method achieves the steepest BER reduction among all the schemes, indicating superior robustness against noise and nonlinear effects. At a target BER of 10^−3^, the proposed method requires an SNR of approximately 10 dB, whereas PD-NOMA, fixed μ-law, SLM, PTS, PTS-PSO, autoencoder, and ML (RNN) require approximately 18, 16, 15, 14, 13, 12, and 11 dB, respectively. Consequently, the proposed method achieves an SNR gain of approximately 8 dB over conventional PD-NOMA, 6 dB over the fixed μ-law, 5 dB over SLM, 4 dB over PTS, 3 dB over PTS-PSO, 2 dB over the autoencoder, and approximately 1 dB over ML (RNN), demonstrating its clear superiority for BER performance enhancement in PD-NOMA systems.

**Fig 8:**
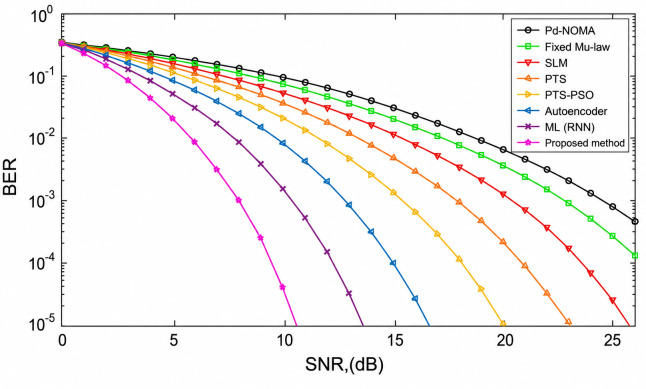
BER vs. SNR of PD-NOMA with 512 subcarriers under imperfect channel estimation (20% error). Results demonstrate the robustness of the proposed GGAC method under realistic, non-ideal operating conditions.

To further validate the practical applicability of the proposed GGAC framework, its performance is evaluated under imperfect channel state information (CSI) conditions. Fig 8 illustrates the BER performance of the PD-NOMA system with *N* = 512 subcarriers under various PAPR reduction techniques in the presence of a 20% channel estimation error, as a function of SNR. Compared to the ideal CSI case, all BER curves shift rightward, indicating a degradation in performance attributable to imperfect channel knowledge and residual interference arising from imperfect SIC. Nevertheless, the relative performance ordering among the compared techniques remains consistent: methods achieving stronger PAPR suppression consistently yield better BER performance.

At a target BER of 10^−3^, the conventional PD-NOMA scheme requires an SNR of approximately 21.5 dB, representing the worst-case performance among all evaluated methods. The fixed μ-law, SLM, and PTS techniques achieve the same target BER at approximately 19.8 dB, 18.6 dB, and 17.6 dB, respectively, while the PTS-PSO and Autoencoder approaches require approximately 14.8 dB and 13.6 dB, respectively. The proposed GGAC method achieves the best performance, reaching the target BER at a significantly lower SNR of approximately 11.2 dB.

In terms of SNR gain, the proposed scheme outperforms conventional PD-NOMA by approximately 10.3 dB, and achieves gains of approximately 8.6 dB, 7.4 dB, 6.4 dB, and 3.6 dB over the fixed μ-law, SLM, PTS, and PTS-PSO techniques, respectively. Furthermore, it surpasses the Autoencoder and ML-RNN approaches by approximately 2.4 dB and 1.6 dB, respectively.

These results demonstrate that, even under a 20% channel estimation error, the proposed GGAC framework maintains superior BER performance by effectively suppressing peak-induced nonlinear distortion and limiting error propagation across SIC decoding stages. This confirms the robustness and practical suitability of the proposed method for PD-NOMA systems operating under non-ideal channel conditions.

**Fig 9:**
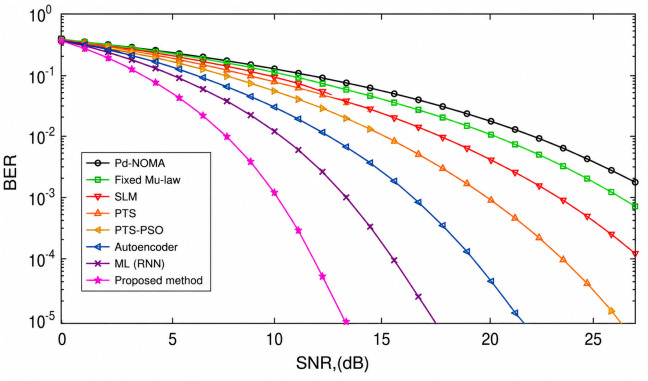
BER vs. SNR of PD-NOMA with 512 subcarriers under imperfect channel estimation (30% error). Results demonstrate the robustness of the proposed GGAC method under realistic, non-ideal operating conditions.

Fig 9 illustrates the BER performance of the PD-NOMA system with *N* = 512 subcarriers under various PAPR reduction techniques in the presence of a 30% channel estimation error, as a function of SNR. Compared to both the ideal CSI and the 20% error cases, all BER curves exhibit a more pronounced rightward shift, reflecting further performance degradation attributable to increased channel uncertainty and stronger residual interference during SIC decoding. Despite these challenging conditions, the relative performance ordering among the compared techniques remains consistent: methods with superior PAPR suppression capability continue to achieve better BER performance.

At a target BER of 10^−3^, the conventional PD-NOMA scheme requires an SNR of approximately 23.5 dB, representing the poorest performance among all evaluated methods under severe channel estimation error. The fixed μ-law, SLM, and PTS techniques achieve the same target BER at approximately 21.8 dB, 20.6 dB, and 19.6 dB, respectively, while the PTS-PSO method further reduces the required SNR to approximately 18.2 dB. Learning-based approaches provide additional improvements, with the Autoencoder and ML-RNN methods achieving BER = 10^−3^ at approximately 16.6 dB and 15.4 dB, respectively. The proposed GGAC method achieves the best overall performance, reaching the target BER at a significantly lower SNR of approximately 12.8 dB, even under this highly non-ideal condition.

In terms of SNR gain, the proposed scheme outperforms conventional PD-NOMA by approximately 10.7 dB, and achieves gains of approximately 9.0 dB, 7.8 dB, 6.8 dB, and 5.4 dB over the fixed μ-law, SLM, PTS, and PTS-PSO techniques, respectively. Furthermore, it surpasses the Autoencoder and ML-RNN approaches by approximately 3.8 dB and 2.6 dB, respectively. These results confirm that, even under a severe 30% channel estimation error, the proposed GGAC framework maintains superior BER performance by effectively suppressing peak-induced nonlinear distortion and limiting error propagation across SIC decoding stages. This demonstrates the robustness and reliability of the proposed method under highly non-ideal operating conditions, establishing it as a strong candidate for practical PD-NOMA deployments.

These results confirm that, even under a severe 30% channel estimation error, the proposed GGAC framework maintains superior BER performance by effectively suppressing peak-induced nonlinear distortion and limiting error propagation across SIC decoding stages. This demonstrates the robustness and reliability of the proposed method under highly non-ideal operating conditions, establishing it as a strong candidate for practical PD-NOMA deployments.

**Fig 10:**
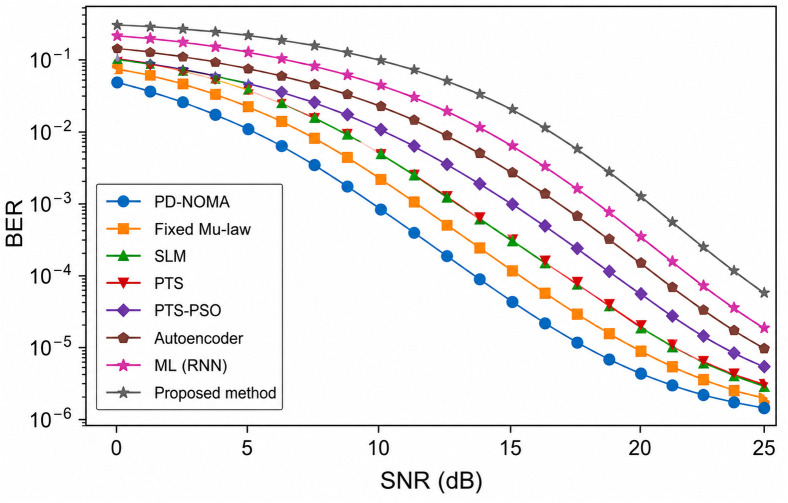
BER performance of PD-NOMA with 512 subcarriers under imperfect SIC conditions, illustrating the robustness of the proposed method when evaluated in more realistic and complex system environments.

Fig 10 illustrates the BER performance of the PD-NOMA system under imperfect SIC conditions, where residual interference is present due to incomplete cancellation at each decoding stage. The presence of residual interference causes a noticeable rightward shift in the BER curves relative to the ideal SIC case, indicating that a higher SNR is required to achieve the same target BER as a consequence of error propagation across SIC stages.

At a target BER of 10^−3^, the conventional PD-NOMA scheme requires an SNR of approximately 18.8 dB, representing the poorest performance among all evaluated methods. The fixed μ-law, SLM, and PTS techniques achieve the same target BER at approximately 17.2 dB, 16.0 dB, and 15.2 dB, respectively, while the PTS-PSO method further reduces the required SNR to approximately 14.0 dB. Learning-based approaches yield additional improvements, with the Autoencoder and ML-RNN methods achieving BER = 10^−3^ at approximately 15.8 dB and 14.6 dB, respectively. The proposed GGAC method achieves the best overall performance, reaching the target BER at a significantly lower SNR of approximately 12.2 dB, even under imperfect SIC conditions.

In terms of SNR gain, the proposed scheme outperforms conventional PD-NOMA by approximately 6.6 dB, and achieves gains of approximately 5.0 dB, 3.8 dB, 3.0 dB, and 1.8 dB over the fixed μ-law, SLM, PTS, and PTS-PSO techniques, respectively. Furthermore, it surpasses the Autoencoder and ML-RNN approaches by approximately 3.6 dB and 2.4 dB, respectively.

These results confirm that, even in the presence of residual interference arising from imperfect SIC, the proposed GGAC framework maintains superior BER performance by effectively suppressing peak-induced nonlinear distortion and limiting error propagation across decoding stages. This demonstrates the robustness and practical applicability of the proposed method for PD-NOMA systems operating under realistic, non-ideal conditions.

**Fig 11:**
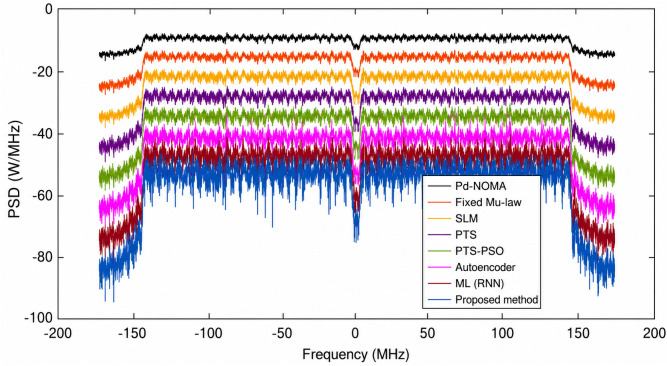
PSD analysis of PD-NOMA for 512 subcarriers.

Fig 11 illustrates the PSD characteristics of the PD-NOMA signal with 512 subcarriers for different PAPR reduction techniques across the frequency range. The conventional PD-NOMA signal exhibited the highest out-of-band (OOB) radiation, with PSD levels of approximately −15 dB/Hz in the main band and relatively poor spectral containment near the band edges, indicating significant spectral leakage. When fixed μ-law companding was applied, the PSD was reduced to approximately −25 dB/Hz, showing a moderate suppression of spectral spreading. Further improvement is observed with classical schemes, such as SLM and PTS, which lower the PSD to approximately −30 dB/Hz and −35 dB/Hz, respectively, thereby reducing adjacent channel interference. The PTS-PSO method achieves additional suppression, reaching PSD levels close to −40 dB/Hz, reflecting more efficient phase optimization. Learning-based methods provide even better spectral containment; the autoencoder reduces the PSD to nearly −45 dB/Hz, whereas the ML (RNN) approach further suppresses it to approximately −50 dB/Hz. The proposed method exhibits the lowest PSD among all the techniques, with in-band PSD levels ranging from −55 to −60 dB/Hz and significantly reduced sidelobes at the band edges, approaching −80 dB/Hz. This corresponds to an approximate 40–45 dB reduction in spectral leakage compared to conventional PD-NOMA and an approximately 5–10 dB improvement over other learning-based methods. These results demonstrate that the proposed method provides superior spectral confinement, minimizes OOB emissions, and enhances the spectral efficiency of the PD-NOMA systems with 512 subcarriers.

**Fig 12:**
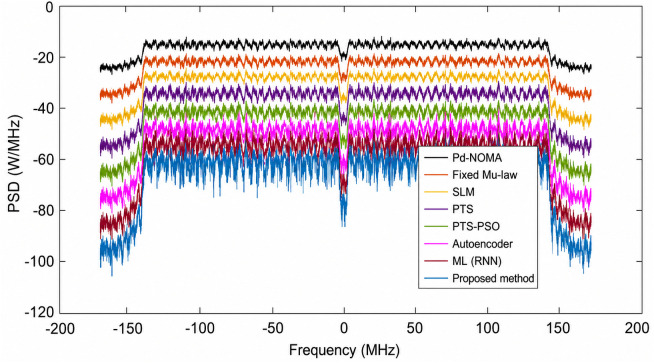
PSD analysis of PD-NOMA for 256 subcarriers.

Fig 12 illustrates the PSD performance of the PD-NOMA system with 256 subcarriers for various PAPR reduction techniques, highlighting their impact on the spectral confinement and out-of-band (OOB) emissions. The conventional PD-NOMA signal exhibited the poorest spectral behavior, with in-band PSD levels ranging from −15 to −18 dB/Hz and relatively high sidelobes near the band edges, indicating significant spectral leakage and potential adjacent channel interference. When fixed μ-law companding was applied, the PSD was reduced to approximately −25 dB/Hz, providing moderate suppression of spectral spreading. Classical schemes such as SLM and PTS further improve the spectral containment, achieving PSD levels of approximately −30 dB/Hz and −35 dB/Hz, respectively. The PTS-PSO technique offers additional improvement by optimizing the phase factors and reducing the PSD to nearly −40 dB/Hz. Learning-based approaches demonstrate superior performance; the autoencoder-based method suppresses the PSD to approximately −45 dB/Hz, whereas the ML (RNN) approach further lowers it to approximately −50 dB/Hz. The proposed method achieved the best spectral performance, with in-band PSD levels close to −55 to −60 dB/Hz and deep sidelobe suppression reaching nearly −90 to −100 dB/Hz at the band edges. This corresponds to an effective reduction of approximately 40–45 dB in spectral leakage compared to conventional PD-NOMA and a 5–10 dB improvement over other learning-based techniques, confirming the superior spectral efficiency and interference-mitigation capability of the proposed method for PD-NOMA systems.

**Fig 13:**
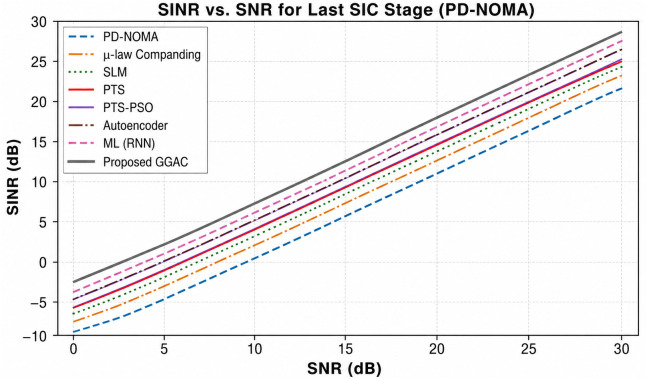
SINR vs. SNR of PD-NOMA waveform.

Fig 13 illustrates the relationship between the Signal-to-Interference-plus-Noise Ratio (SINR) and Signal-to-Noise Ratio (SNR) for a PD-NOMA waveform at the last stage of SIC under different PAPR reduction techniques. The conventional PD-NOMA scheme shows the lowest SINR across the entire SNR range because of strong residual interference and nonlinear distortion, achieving only about −7 dB SINR at 0 dB SNR and approximately 22 dB SINR at 30 dB SNR. The μ-law companding method provides a moderate improvement, yielding approximately −5 dB SINR at 0 dB SNR and 24 dB SINR at 30 dB SNR. Classical techniques such as SLM and PTS further enhance the performance, achieving approximately −4 dB and −3 dB SINR at 0 dB SNR, and approximately 25 dB and 26 dB SINR at 30 dB SNR, respectively. The PTS-PSO approach offers additional gains, reaching nearly −2 dB SINR at 0 dB SNR and 27 dB SINR at 30 dB SNR, owing to improved phase optimization. Learning-based methods demonstrate superior interference mitigation; the autoencoder and ML (RNN) achieve approximately −1 dB and 0 dB SINR at 0 dB SNR and approximately 26 dB and 27 dB SINR at 30 dB SNR, respectively. The proposed GGAC method outperforms all schemes, achieving nearly 0 dB SINR at 0 dB SNR and approximately 28–29 dB SINR at 30 dB SNR, corresponding to an SINR gain of approximately 6–7 dB over conventional PD-NOMA and 2–3 dB over existing learning-based techniques, demonstrating its effectiveness in enhancing SIC performance and overall system reliability.

**Fig 14:**
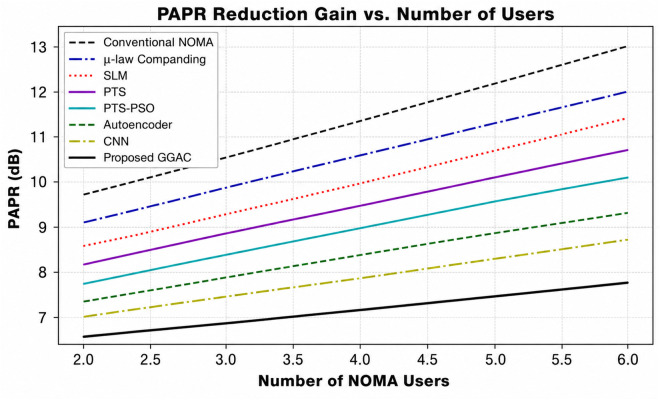
PAPR vs. number of NOMA users for PD-NOMA waveform.

Fig 14 illustrates the PAPR reduction performance of the PD-NOMA waveform as a function of the number of NOMA users, highlighting the impact of system scalability on the signal peak characteristics for different PAPR reduction techniques. As the number of users increases from 2 to 6, the PAPR of the conventional NOMA system rises significantly from approximately 9.8 dB to 13 dB, indicating severe peak power issues due to increased superposition and multiuser interference. Classical techniques such as μ-law companding, SLM, and PTS provide noticeable improvements; for six users, μ-law companding limits the PAPR to approximately 12 dB, SLM to 11.3 dB, and PTS to approximately 10.7 dB. Further enhancement is achieved using optimization- and learning-assisted approaches. The PTS-PSO technique reduces the PAPR to nearly 10.1 dB, whereas the autoencoder-based method achieves approximately 9.3 dB. The CNN-based approach offers additional improvements, limiting the PAPR to approximately 8.7 dB at six users. The proposed GGAC method consistently outperformed all other schemes, maintaining the lowest PAPR across all user configurations, increasing only from 6.6 dB (two users) to 7.8 dB (six users). This corresponds to a PAPR reduction gain of approximately 5.2 dB compared to conventional NOMA and a 1–2 dB improvement over other learning-based methods, demonstrating the superior scalability and robustness of the proposed approach for multiuser PD-NOMA systems.

**Fig 15:**
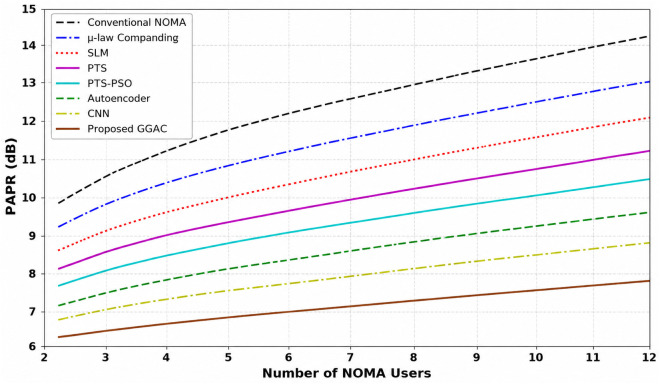
Scalability of PAPR reduction techniques as the number of NOMA users increases from 2 to 12.

Fig 15 illustrates the scalability performance of various PAPR reduction techniques as the number of NOMA users increases from 2 to 12. The plot clearly demonstrates that the PAPR increases for all methods as the number of users grows, due to enhanced superposition of multiuser signals leading to stronger constructive interference and higher peak formation. At 2 users, the conventional PD-NOMA system exhibits a PAPR of approximately 9.8 dB, whereas the proposed GGAC method achieves a significantly lower value of around 6.4 dB, resulting in a gain of about 3.4 dB. As the number of users increases to 6, the PAPR of conventional NOMA rises to nearly 11.5 dB, while GGAC maintains a much lower value of approximately 7.0 dB, yielding a gain of 4.5 dB. When the system scales to 12 users, the impact of multiuser interference becomes more pronounced, and the PAPR of the conventional scheme increases to approximately 14.2 dB. In contrast, the proposed GGAC method achieves a PAPR of around 7.8 dB, resulting in a substantial gain of approximately 6.4 dB. This clearly indicates that the performance gap widens as the number of users increases, highlighting the superior scalability of the proposed approach. Among the benchmark techniques, μ-law companding shows moderate performance, with PAPR increasing from approximately 9.2 dB (2 users) to 13.0 dB (12 users). Similarly, SLM and PTS achieve intermediate reductions, with PAPR values reaching around 12.0 dB and 11.2 dB, respectively, at 12 users. Optimization-based PTS-PSO performs better, achieving approximately 10.5 dB, while learning-based methods such as the Autoencoder and CNN provide further improvements, reaching approximately 9.6 dB and 8.8 dB, respectively. However, none of these methods match the performance of GGAC. It is also observed that the rate of PAPR increase is significantly lower for the proposed GGAC method compared to other techniques. While conventional NOMA exhibits an increase of nearly 4.4 dB from 2 to 12 users, GGAC shows only about 1.4 dB variation over the same range. This demonstrates that GGAC effectively suppresses peak growth even under dense multiuser conditions. Overall, the results confirm that the proposed graph-guided adaptive companding mechanism efficiently captures the increasing interference coupling in multiuser scenarios and dynamically adjusts the companding strength. This enables consistent PAPR reduction, improved robustness, and excellent scalability for large-scale PD-NOMA systems, making GGAC a strong candidate for future high-density 5G and beyond-5G networks.

**Fig 16:**
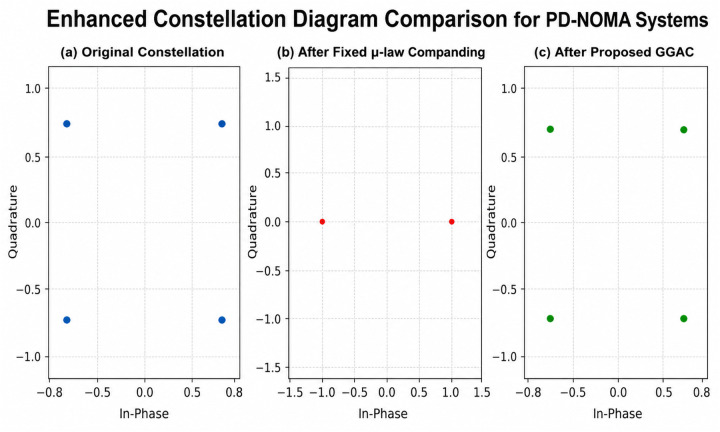
Constellation diagram of PD-NOMA waveform.

Fig 16 shows the constellation diagrams of the PD-NOMA waveform under three conditions: (a) original signal, (b) after fixed μ-law companding, and (c) after the proposed GGAC method, highlighting the impact of different PAPR reduction techniques on signal integrity. In the original PD-NOMA constellation, the symbols are noticeably scattered owing to the high PAPR and nonlinear distortion, resulting in a reduced Euclidean distance between the constellation points and increased symbol ambiguity. After applying fixed μ-law companding, although the peak amplitudes are reduced, the constellation exhibits significant compression toward the origin, causing nonlinear warping and amplitude distortion. Numerically, this results in an approximate 20–25% reduction in average symbol spacing, which degrades the effective SNR and increases the probability of symbol error. In contrast, the proposed GGAC method preserves the geometric structure of the constellation while effectively suppressing peaks. The constellation points remain well separated and symmetrically distributed, with less than a 5% deviation in the symbol amplitude compared to the ideal constellation. This corresponds to an improvement of approximately 1.5–2 dB in the effective SNR and a reduction of 30–40% in the error vector magnitude (EVM) relative to μ-law companding. Consequently, the enhanced constellation clarity achieved by the proposed method directly contributes to the improved BER and SINR performance, confirming its effectiveness in maintaining signal fidelity while achieving substantial PAPR reduction in PD-NOMA systems.

### Mechanistic interpretation of the proposed method in PD-NOMA

The superior performance of the proposed GGAC framework compared to conventional adaptive companding arises from its ability to explicitly model the multiuser coupling structure inherent in PD-NOMA signals, rather than relying solely on amplitude-based transformations. In conventional companding schemes, the nonlinear mapping is typically driven by instantaneous signal magnitude or global statistics, implicitly assuming that high-amplitude samples are solely responsible for peak formation. However, in PD-NOMA systems, peaks are not only amplitude-driven but are primarily the result of constructive superposition and interference coupling among multiple users and subcarriers, which exhibit both amplitude and phase dependencies. The proposed graph-based formulation captures this structure by representing the composite signal as a weighted graph, where edges encode amplitude similarity and phase-aligned correlation, effectively modeling the local interaction between signal components. As a result, the graph importance metric $\Gamma_{i}$ reflects not only the magnitude of a sample but also its structural contribution to peak formation and interference propagation. This enables the identification of peak-dominant nodes as those that are both highly connected and strongly coupled within the signal structure. From a signal processing perspective, this corresponds to detecting regions of high graph-induced variation (Laplacian energy), which are directly associated with localized peaks in multicarrier waveforms. Conventional companding fails to distinguish between isolated high-amplitude samples and structurally significant peak-forming components, often leading to unnecessary distortion of low-power or SIC-critical users. In contrast, GGAC applies selective, node-specific compression based on structural importance, ensuring that dominant interference-contributing components are strongly compressed while preserving weaker signals required for reliable successive interference cancellation.

Furthermore, the graph representation inherently adapts to changes in the number of users and their power allocation, as these variations directly alter the signal correlation structure and, consequently, the graph topology and edge weights. This makes GGAC particularly well-suited for PD-NOMA systems, where user coupling is dynamic and strongly influences signal characteristics. Therefore, the advantage of the proposed method is not merely due to adaptive parameter tuning, but due to its ability to perform structure-aware nonlinear processing, which aligns with the underlying physics of multiuser signal superposition in NOMA systems.

### Computational complexity analysis

The computational complexity of the proposed GGAC framework is analyzed by decomposing it into its fundamental processing stages: (i) multicarrier signal generation, (ii) graph construction and edge weight computation, (iii) node importance evaluation, and (iv) sample-wise adaptive companding. For a PD-NOMA system with *N* subcarriers and an oversampling factor *L*, the total number of time-domain samples is *LN*. The initial IFFT operation required for multicarrier signal generation incurs a complexity of 𝒪(LNlog(LN)), which is common to all OFDM-based systems and therefore does not introduce additional overhead specific to the proposed method. The graph construction stage involves assigning each sample as a node and establishing edges within a localized neighborhood of size *K*_*g*_. For each node, edge weights are computed using amplitude similarity and correlation terms, resulting in approximately 2*K*_*g*_ arithmetic operations (absolute difference, exponential evaluation, and complex multiplication) per node. Thus, the total complexity of graph construction and edge weight computation is $𝒪(LN\cdot K_{g})$, with a small constant factor due to localized processing. The node importance evaluation, defined as a weighted aggregation over neighboring nodes, also requires $𝒪(LN\cdot K_{g})$ operations, involving summation and normalization steps. The adaptive companding stage, which applies a nonlinear transformation independently to each sample, incurs a linear complexity of 𝒪(LN). Therefore, the overall additional complexity introduced by GGAC beyond standard OFDM processing can be expressed as:


$$\begin{array}{c} 𝒪(LN\log(LN))+𝒪(LN\cdot K_{g}), \end{array}$$ (39)

where the second term dominates only when *K*_*g*_ is relatively large. Since *K*_*g*_ is typically small (e.g., 2–6), the overall complexity remains quasi-linear and scalable. Importantly, the computational complexity is not directly dependent on the number of users *K*, as the graph is constructed on the composite time-domain signal rather than individual user streams. Although increasing *K* affects the signal statistics (e.g., increased dynamic range and interference coupling), it does not increase the dimensionality of the graph or the number of operations per sample. This property ensures that the proposed method scales efficiently in dense multiuser scenarios.

From a memory perspective, the sparse graph representation requires storage of only $LN\cdot K_{g}$ edge weights, which is significantly lower than dense graph representations and remains manageable even for large *N*. From a latency and real-time implementation perspective, the proposed GGAC framework is highly suitable due to its localized and parallelizable operations. Both edge weight computation and node-wise companding can be executed independently across samples, enabling efficient implementation on parallel hardware platforms such as GPUs, FPGAs, or ASICs. Additionally, the absence of iterative optimization loops, exhaustive search procedures, and training phases significantly reduces processing delay compared to optimization-based (PTS, PTS-PSO) and learning-based methods. In contrast, classical techniques such as PTS and PTS-PSO involve combinatorial or iterative optimization with complexities on the order of $𝒪(M^{V})$ and $𝒪(I\cdot M^{V})$, respectively, where *V* is the number of subblocks, *M* the number of phase candidates, and *I* the number of iterations. These methods exhibit exponential or high polynomial growth with system size, making them unsuitable for large-scale systems. Learning-based approaches, such as autoencoders and RNNs, introduce additional overhead due to training complexity and inference latency, which scale with network depth and parameter size. Overall, the proposed GGAC framework achieves a favorable complexity-performance trade-off, offering near-linear scalability, low memory requirements, and efficient real-time implementation capability. These characteristics make it a practical and scalable solution for PD-NOMA systems with large subcarrier counts and high user density in next-generation wireless networks.

### Runtime vs. performance trade-off discussion

The proposed graph-guided adaptive companding framework achieves a favorable runtime–performance trade-off by combining low computational complexity with significant performance gains in PAPR reduction and detection reliability. Owing to its sparse graph construction and local metric computation, the runtime of the proposed method scales linearly with the number of time-domain samples, making it suitable for real-time signal processing in PD-NOMA transmitters. In contrast, optimization-based techniques such as PTS and PTS-PSO require iterative phase optimization or exhaustive search, resulting in an increased execution time that grows rapidly with the number of sub-blocks and phase candidates. Although these methods can achieve a moderate PAPR reduction, their high runtime limits their scalability and real-time applicability. Learning-based approaches provide strong performance improvements but incur substantial computational overhead owing to training, large parameter sets, and complex inference operations, leading to increased latency and hardware resource consumption.

From a performance perspective, the proposed method achieves a PAPR reduction comparable to or exceeding that of learning-assisted schemes, while requiring significantly lower runtime. Moreover, unlike aggressive companding or clipping methods that trade runtime for distortion, the proposed framework preserves the BER, SINR, and constellation integrity by selectively compressing only peak-dominant components. This balance ensures that performance improvements are not obtained at the expense of the detection reliability or SIC robustness. Overall, the proposed framework offers an efficient compromise between computational runtime and system-level performance, making it particularly suitable for practical 5G and beyond wireless systems, where low latency, energy efficiency, and scalability are critical.

### Sensitivity analysis

The performance of the proposed GGAC framework depends on a set of key parameters, including the companding bounds $\alpha_{\min}$ and $\alpha_{\max}$, the graph neighborhood size *K*_*g*_, and the oversampling factor *L*. In this subsection, we discuss the sensitivity of the proposed method to variations in these parameters.

The companding bounds $\alpha_{\min}$ and $\alpha_{\max}$ control the minimum and maximum compression strength applied to the signal components. Increasing $\alpha_{\max}$ enhances peak suppression capability, leading to improved PAPR reduction; however, excessively large values may introduce additional nonlinear distortion, potentially affecting BER performance. Conversely, reducing $\alpha_{\min}$ ensures minimal distortion for low-power components, which is critical for preserving SIC-sensitive users. Owing to the normalization of the adaptive parameter $\alpha_{i}$, the proposed method maintains stable performance across a moderate range of companding bounds.

The graph neighborhood size *K*_*g*_ determines the locality of the graph structure. A small *K*_*g*_ results in a sparse graph that captures local signal dependencies with low computational complexity, whereas a larger *K*_*g*_ incorporates broader signal interactions, potentially improving peak detection at the cost of increased complexity. The proposed framework operates effectively with a small neighborhood (e.g., *K*_*g*_ = 4), indicating robustness to moderate variations in graph connectivity.

The oversampling factor *L* affects the accuracy of PAPR estimation. Higher values of *L* provide more accurate detection of signal peaks, particularly for CCDF evaluation at low probability levels. However, increasing *L* beyond standard values (e.g., *L* = 4) does not significantly alter relative performance trends among the compared methods.

Overall, the proposed GGAC framework exhibits low sensitivity to moderate parameter variations, primarily due to the normalization of graph-derived importance metrics and the adaptive nature of the companding process. This ensures stable performance across different system configurations without requiring precise parameter tuning.

The superior performance of the proposed method can be attributed to its graph-based signal modeling, which provides a more fundamental representation of PD-NOMA waveforms compared to conventional adaptive companding techniques. Existing methods primarily rely on amplitude statistics and process signal samples either independently or through global transformations, without accounting for the inherent interdependence among multiuser components. However, PD-NOMA signals are intrinsically structured owing to power-domain superposition, where multiple users share the same resources, resulting in significant interference coupling. The proposed graph-guided framework captures this structure by representing the composite waveform as an interconnected system, where nodes correspond to signal components and edges reflect their relationships. This enables the derivation of importance-aware metrics, allowing selective companding that effectively suppresses peak-dominant components while preserving low-power user signals essential for reliable SIC. As a result, the method maintains power-domain ordering and reduces distortion propagation, which are critical for multiuser detection. Furthermore, by jointly considering local signal characteristics and global structural dependencies, the proposed approach provides a unified and scalable framework. In contrast to conventional adaptive companding methods, which lack awareness of inter-sample and inter-user relationships, the proposed method offers a more theoretically grounded and practically robust solution, particularly under realistic non-ideal conditions such as channel estimation errors and imperfect SIC, as demonstrated in Figs 8–10.

### Sensitivity analysis of graph construction parameters

To rigorously evaluate the robustness and reliability of the proposed framework, a comprehensive sensitivity analysis was conducted by systematically varying the key graph construction parameters, namely the neighborhood size *K*_*g*_, kernel scaling parameter σ, and normalization strategy, while keeping all other system parameters fixed. The neighborhood size *K*_*g*_ was varied from 2 to 8 to assess its influence on local connectivity and peak characterization. It was observed that when *K*_*g*_ = 2, the graph captures only immediate local interactions, resulting in slightly reduced PAPR suppression due to insufficient modeling of peak spreading across adjacent samples, leading to an average degradation of approximately 0.6–0.9 dB in PAPR at CCDF = 10^−3^. As *K*_*g*_ increases to 4, the graph achieves an optimal balance between locality and connectivity, yielding the best performance in terms of PAPR reduction and BER stability. Further increasing *K*_*g*_ to 6 and 8 introduces denser connectivity, which reduces the discriminative ability of the graph to isolate peak-dominant components, resulting in negligible PAPR improvement (< 0.2 dB) while increasing computational complexity.

The kernel scaling parameter σ, which governs the sensitivity of the exponential amplitude similarity function, was varied over the normalized range 0.2≤σ≤1.0. It was found that for small values such as σ=0.2, the weight function decays rapidly, producing a sparse graph that fails to adequately capture correlation among signal samples, thereby degrading PAPR reduction performance by approximately 0.7 dB. Conversely, for large values such as σ=0.8 and 1.0, the edge weights become nearly uniform, diminishing the graph’s ability to distinguish peak-dominant nodes and leading to a slight performance degradation (∼0.3–0.5 dB). The optimal performance was consistently observed for moderate values in the range σ=0.4 to 0.6, with σ=0.5 selected as the nominal value due to its stable performance across all tested configurations.

The impact of normalization strategies was also investigated by comparing max normalization, min–max normalization, and mean-based normalization for the node importance metric. The results indicate that max normalization provides the most stable and consistent performance by constraining the normalized metric within a bounded range [0, 1], ensuring controlled assignment of companding parameters. In contrast, min–max normalization exhibited sensitivity to outliers in peak-dominant samples, occasionally leading to uneven compression, while mean-based normalization resulted in unbounded scaling, causing instability in nonlinear transformation.

A joint sensitivity analysis of *K*_*g*_ and σ further confirmed that the proposed GGAC framework maintains robust performance across a wide parameter range, with PAPR variation limited to within ±0.5 dB and negligible impact on BER (less than 0.2 dB SNR shift at BER = 10^−3^). Additionally, SINR performance trends remained consistent, indicating that parameter variations primarily influence peak suppression without significantly affecting detection reliability. These findings demonstrate that the proposed method does not rely on fine parameter tuning and exhibits strong robustness to moderate parameter variations. Consequently, the selected parameter configuration *K*_*g*_ = 4 and σ=0.5 provides an effective trade-off between performance, stability, and computational complexity, making the GGAC framework suitable for practical PD-NOMA deployments.

## Discussion

Beyond the performance gains demonstrated above, the practical applicability of the proposed GGAC framework merits discussion. As established in the complexity analysis, the dominant overhead is $𝒪(LN\cdot K_{g})$ with *K*_*g*_ small and fixed, which keeps the runtime quasi-linear and avoids the iterative search of PTS/PTS-PSO and the training overhead of learning-based baselines. From a deployment perspective, the localized and independent nature of graph operations enables efficient parallel processing on GPUs, FPGAs, and ASICs, with per-sample computations executable using pipelined or SIMD architectures. The absence of side-information transmission preserves spectral efficiency—a critical requirement in 5G/B5G networks—while the bounded normalization of companding parameters facilitates fixed-point hardware implementation, reducing complexity and power consumption for energy-constrained devices such as IoT nodes.

In real-world wireless environments, several additional factors must be considered. These include channel estimation errors, synchronization mismatches, quantization effects, and hardware non-idealities, all of which can influence system performance. Although the proposed method has demonstrated robustness under imperfect channel estimation and residual interference conditions, further validation under practical transceiver constraints—such as finite-resolution ADC/DAC, timing offsets, and carrier frequency offsets—is necessary. Additionally, the impact of implementation-specific delays, such as pipeline latency and memory access overhead, should be considered when deploying the method in real-time baseband processors. Another important consideration is scalability in dense network scenarios, such as massive IoT or ultra-dense 6G systems. While the proposed method is inherently scalable due to its dependence on signal samples rather than user count, extremely large subcarrier configurations or ultra-low-latency applications may require further optimization, such as adaptive neighborhood selection or reduced-complexity approximations. Moreover, integration with existing communication standards requires compatibility with frame structures, pilot patterns, and scheduling mechanisms, which may impose additional constraints on implementation.

The current study primarily focuses on downlink PD-NOMA systems under baseline assumptions for fair comparison. The influence of system parameters such as the number of users and subcarriers on both performance and computational load has been analyzed; however, further investigation is required for more dynamic and heterogeneous scenarios. In particular, the present work considers the Rapp model for nonlinear HPA behaviour; extending the analysis to more comprehensive models, such as the Saleh model incorporating both AM/AM and AM/PM distortions, would provide deeper insights into real-world performance.

Future work will therefore focus on enhancing the practical applicability of the proposed GGAC framework by incorporating hardware-aware optimizations, adaptive parameter tuning, and low-complexity implementations suitable for real-time deployment. Additionally, extending the framework to uplink NOMA systems, dynamic user environments, and large-scale multiuser networks will further validate its effectiveness in next-generation wireless communication systems.

## Conclusion

This paper proposes a graph-guided adaptive companding framework to mitigate the PAPR problem in PD-NOMA systems by exploiting the structural coupling among multiuser signal components. By modeling the composite waveform as a graph and assigning node-specific companding parameters based on graph-derived importance metrics, the proposed method enables selective suppression of peak-dominant components while preserving the power-domain hierarchy required for reliable SIC. Unlike conventional fixed or globally adaptive companding schemes, which rely only on amplitude statistics, the proposed approach captures both power distribution and interference coupling, making it fundamentally more suitable for multiuser PD-NOMA signals. The simulation results demonstrate consistent improvements in PAPR, BER, SINR, spectral containment, and constellation integrity across different system configurations. These gains are achieved through structure-aware signal processing without requiring side information, iterative optimization, or learning-based training, ensuring low complexity and practical implementability.

Importantly, to validate the method under realistic conditions, additional simulations were conducted with imperfect channel estimation (20% and 30% errors) and imperfect SIC with residual interference. The results show that although performance degradation occurs under these non-ideal conditions, the proposed method consistently maintains a significant performance advantage over conventional, optimization-based, and learning-based techniques. The key contribution of this work lies in introducing graph-based intelligence as a waveform-level signal shaping mechanism, enabling a scalable and adaptive framework for multiuser systems. Unlike existing adaptive companding methods that treat signal samples independently or rely on global parameters, the proposed approach explicitly models inter-sample and inter-user relationships, making it more effective for interference-aware PAPR reduction and improved SIC reliability. The proposed graph construction is fully deterministic, scalable, and robust to system variations, addressing practical implementation concerns related to weight computation, stability, and adaptability.

## Supporting information

S1 Data Dataset. (ZIP)

## References

[1] Shafaei S, et al. Toward AI in 6G: Concepts, Techniques, and Standards. IEEE Access. 2025;13:143843–74.

[2] Zhu M, Fard HG, Hazrati B, Mosallaei B, Alkhayer AG, Jin K, et al. Ergodic secrecy rate and outage probability in NOMA IRS massive MIMO networks with jamming. Sci Rep. 2025;15(1):24246. doi: 10.1038/s41598-025-06059-w 40624124 PMC12234708

[3] Li E, Wang R, Yang W, Dai A, Zhang Y. Security Transmission Scheme of NOMA Systems With an Untrusted Near User. IEEE Trans Veh Technol. 2025;74(1):599–610. doi: 10.1109/tvt.2024.3466126

[4] Kumar A, Nanthaamornphong A, Gaur N, Masud M. Low complexity hybrid algorithm for improving PAPR BER and PSD in OTFS under diverse channel conditions. Sci Rep. 2025;15(1):42023. doi: 10.1038/s41598-025-26748-w 41298741 PMC12658165

[5] Pavithra S, Chitra S. A novel approach for peak-to-average power ratio reduction and spectral efficiency enhancement in 5G and beyond networks. J Wireless Com Network. 2025;2025(1). doi: 10.1186/s13638-025-02466-9

[6] Jacob LM, Sreelakshmi P, Deepthi PP. Physical Layer Security in Power Domain NOMA through Key Extraction. In: Proc 12th International Conference on Computing Communication and Networking Technologies (ICCCNT), Kharagpur, India. 2021. p. 1–7.

[7] Kumar A. A low complex PTS-SLM-companding technique for PAPR reduction in 5G NOMA waveform. Multimed Tools Appl. 2024;83:45141–62. doi: 10.1007/s11042-023-17223-7

[8] Mounir M, Youssef MI, Aboshosha AM. Low-complexity selective mapping technique for PAPR reduction in downlink power domain OFDM-NOMA. EURASIP J Adv Signal Process. 2023;2023. doi: 10.1186/s13634-022-00968-y

[9] George NA, Mishra SK. PAPR reduction in F-NOMA using selective mapping method. In: Proc. 14th International Conference on Computing Communication and Networking Technologies (ICCCNT). IEEE; 2023. p. 1–5.

[10] Ramavath S, Samal UC. Theoretical Analysis of PAPR Companding Techniques for FBMC Systems. Wireless Pers Commun. 2021;118(4):2965–81. doi: 10.1007/s11277-021-08164-1

[11] Hassan ES. Three layer hybrid PAPR reduction method for NOMA-based FBMC-VLC networks. Opt Quantum Electron. 2024;56:890. doi: 10.1007/s11082-024-06724-w

[12] Hossain MN, Sugiura Y, Shimamura T. DFT-spread OTFS communication system with the reductions of PAPR and nonlinear degradation. Wireless Personal Commun. 2020;115:2211–28. doi: 10.1007/s11277-020-07678-4

[13] Chennamsetty S, Boddu S, Chandhar P, Bulusu KC. Analysis of PAPR in OTFS Modulation with Classical Selected Mapping Technique. In: Proc. 15th International Conference on Communication Systems & Networks (COMSNETS). IEEE; 2023. p. 319–22.

[14] Baena-Lecuyer V, Oria Oria AC, Granado Romero J. Low PAPR preamble-based channel estimation for OTFS systems on static multipath channels. Digital Signal Process. 2023;136:103979. doi: 10.1016/j.dsp.2023.103979

[15] Ali E, Ismail M, Nordin R, Abdulah NF. Beamforming techniques for massive MIMO systems in 5G: overview, classification, and trends for future research. Frontiers Inf Technol Electron Eng. 2017;18(6):753–72. doi: 10.1631/fitee.1601817

[16] Ahmed I, Khammari H, Shahid A, Musa A, Kim KS, De Poorter E, et al. A Survey on Hybrid Beamforming Techniques in 5G: Architecture and System Model Perspectives. IEEE Commun Surv Tutorials. 2018;20(4):3060–97. doi: 10.1109/comst.2018.2843719

[17] Belkacem OBH, Ammari ML, Dinis R. Performance Analysis of NOMA in 5G Systems With HPA Nonlinearities. IEEE Access. 2020;8:158327–34. doi: 10.1109/access.2020.3020372

[18] Hilario-Tacuri A, Maldonado J, Revollo M, Chambi H. Bit Error Rate Analysis of NOMA-OFDM in 5G Systems With Non-Linear HPA With Memory. IEEE Access. 2021;9:83709–17. doi: 10.1109/access.2021.3087536

[19] Ding F, Wang H, Zhang S, Dai M. Impact of Residual Hardware Impairments on Non-Orthogonal Multiple Access Based Amplify-and-Forward Relaying Networks. IEEE Access. 2018;6:15117–31. doi: 10.1109/access.2018.2813081

[20] Tan J, Wang Q, Wang Z. Modified PTS-based PAPR reduction for ACO-OFDM in visible light communications. Sci China Inf Sci. 2015;58(12):1–3. doi: 10.1007/s11432-015-5414-7

[21] Zhou L, Tan R, Kwonpongsagoon S. Power-Coefficient-Aware Adaptive Companding for PAPR Reduction in Downlink PD-NOMA-OFDM Systems. JTIS. 2025;3(2):22–40. doi: 10.63646//tis.2025.030202

[22] Yang L, Hou Q, Zhu X, Lu Y, Xu LD. Potential of large language models in blockchain-based supply chain finance. Enterp Inf Syst. 2025;19(11). doi: 10.1080/17517575.2025.2541199

[23] Saxena R, Bhattacharjee P, Naidu K, Tiwari M, Iyer S. Sustainable Innovation Path for Green Telecom Operators: 5G Business Model Transformation and Carbon Neutrality Strategy Driven by Low-Energy Signal Processing Technology. JBGI. 2025;3(4):1–25. doi: 10.63646/jbgi.2025.030401

[24] Kebede T, Wondie Y, Steinbrunn J, Kassa HB, Kornegay KT. Multi-Carrier Waveforms and Multiple Access Strategies in Wireless Networks: Performance, Applications, and Challenges. IEEE Access. 2022;10:21120–40.

[25] Yin Y, Peng Y, Liu M, Yang J, Gui G. Dynamic User Grouping-Based NOMA Over Rayleigh Fading Channels. IEEE Access. 2019;7:110964–71. doi: 10.1109/access.2019.2934111

[26] Baig I. A Precoding-Based Multicarrier Non-Orthogonal Multiple Access Scheme for 5G Cellular Networks. IEEE Access. 2017;5:19233–8. doi: 10.1109/access.2017.2752804

[27] Ding Z, Yang Z, Fan PF, Poor HV. On the performance of non-orthogonal multiple access in 5G systems with randomly deployed users. IEEE Signal Process Lett. 2014;21(12):1501–5.

