# Supplementary material for: Graph-guided adaptive companding for PAPR reduction in power-domain NOMA systems
Source: PLoS One. 2026 May 21;21(5):e0349671. doi: 10.1371/journal.pone.0349671 (PMC13193346; doi:10.1371/journal.pone.0349671)
Supplement: S1 Data — (ZIP) [file pone.0349671.s001.zip › dataset/data-a.docx]

Supplementary Material: Detailed Simulation, Equations, and Algorithmic Framework for GGAC

**. Complete Specification of Simulation Parameters**

To ensure reproducibility and transparency, all simulation parameters and implementation details of the proposed GGAC framework are explicitly defined in this section. The simulations were conducted using MATLAB -2024-a, and all results were averaged over independent Monte Carlo realizations to ensure statistical reliability.

| **Parameter** | **Value** |
| --- | --- |
| Simulation tool | MATLAB (2025a) |
| Number of users (K) | 2–4 |
| Subcarriers (N) | 128, 256, 512 |
| Modulation | QPSK, 16-QAM |
| Power allocation | (0.6, 0.3, 0.1) |
| Channel model | Rayleigh fading + AWGN |
| Oversampling factor (L) | 4 |
| Monte Carlo runs | (10^4^) |
| Neighborhood size (($K_{g}$ )) | 4 |
| Companding range | $\alpha_{min}=0.5$, $\alpha_{max}=5$ |
| SIC type | Perfect / Imperfect (specified cases) |
| Channel estimation error | 0%, 20%, 30% |
| Performance metrics | PAPR (CCDF), BER, SINR, PSD |

# 1. Signal Model

The transmitted PD-NOMA signal is constructed as:
$s\left[ n \right]= \Sigma_{\left\{ k=1 \right\}}^{\left\{ K \right\}} \sqrt{P_{k}}x_{k}[n]$ where $x_{k\left[ n \right]}$ represents the modulated symbols of user k and $P_{k}$ denotes power allocation.
Power normalization ensures $E[\left| x_{k}\left[ n \right] \right|^{2}] = 1.$

# 2. OFDM Processing

The time-domain signal is obtained via IFFT:
s[n] = IFFT{X[k]}
Oversampling is applied using zero-padding with factor L = 4.

# 3. Graph Construction

Each time-domain sample is modeled as a node.
Edges are defined within neighborhood K_g:

$$|i - j| \leq K_{g}W_{ij}= exp(-\left| \left| s\left[ i \right] \right|- \left| s\left[ j \right] \right| \right| / \sigma) \times\rho_{ij}$$

Correlation term:
$\rho_{ij} = (s[i] s[j]*) / (|s[i]| |s[j]|)$
Graph Laplacian:
L = D - W

# 4. Node Importance Metric

$\Gamma_{i} = \Sigma_{\left\{ j \in N\left( i \right) \right\}} W_{ij}$
Normalized importance:
$\tilde{\Gamma}_{i} = \Gamma_{i} / max(\Gamma)$

# 5. Adaptive Companding

Companding parameter:

$$\alpha_{i} = \alpha_{min} + \left( \alpha_{max} - \alpha_{min} \right)\tilde{\Gamma}_{i}$$

Companding function:
$s_{c\left[ i \right]} = sign(s[i]) \times log(1 + \alpha_{i} |s[i]|) / log(1 + \alpha_{i})$

# 6. Channel Model

Rayleigh fading channel:
h ~ CN(0,1)

Received signal:

$$y[n] = h s_{c\left[ n \right]} + w[n]$$

where w[n] is AWGN noise.

# 7. SIC Receiver

Successive interference cancellation is performed in descending power order.
Residual interference is modeled as:
$y = s - ŝ + \varepsilon s$

# 8. Performance Metrics

PAPR:
$PAPR = max \left| s\left[ n \right] \right|^{2} / E[\left| s\left[ n \right] \right|^{2}]$

Laplacian Energy:
$E_{G} = 1/2 \Sigma W_{ij} \left( s\left[ i \right]- s\left[ j \right] \right)^{2}$
BER and SINR are computed after SIC decoding.

# 9. Algorithm: GGAC Framework

Step 1: Generate modulated symbols
Step 2: Apply PD-NOMA superposition
Step 3: Perform IFFT
Step 4: Construct graph (compute W_ij)
Step 5: Compute Γ_i
Step 6: Normalize Γ_i → Γ̃_i
Step 7: Compute α_i
Step 8: Apply companding
Step 9: Transmit through channel
Step 10: Perform inverse companding and SIC
Step 11: Evaluate PAPR, BER, SINR
