# Supplementary figures and images for: Graph-guided adaptive companding for PAPR reduction in power-domain NOMA systems
Source: PLoS One. 2026 May 21;21(5):e0349671. doi: 10.1371/journal.pone.0349671 (PMC13193346; doi:10.1371/journal.pone.0349671)

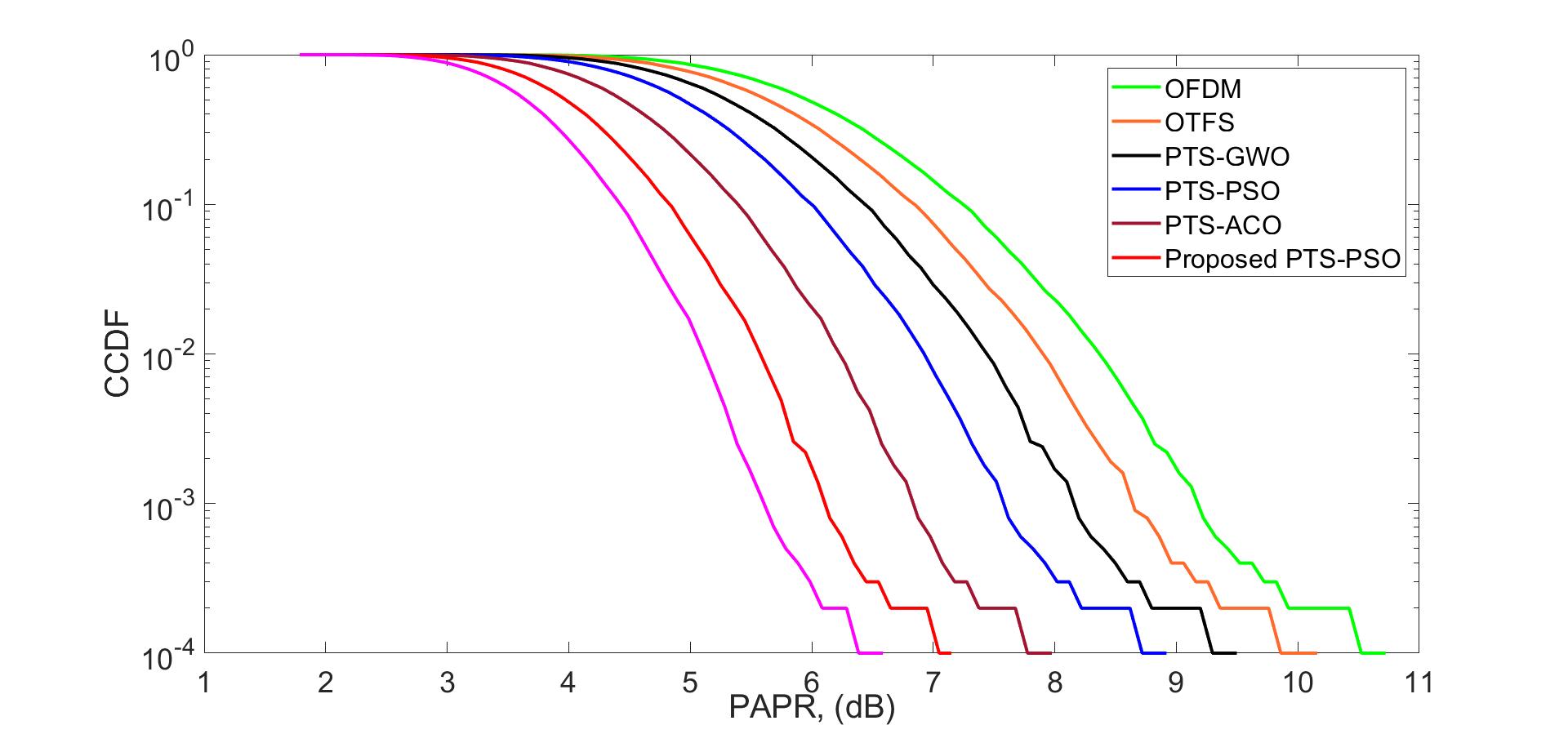

Supplement: S1 Data — (ZIP) [file pone.0349671.s001.zip › dataset/fig 3 64 sub.jpg]
